# Scarless wound healing programmed by core-shell microneedles

**DOI:** 10.1038/s41467-023-39129-6

**Published:** 2023-06-10

**Authors:** Ying Zhang, Shenqiang Wang, Yinxian Yang, Sheng Zhao, Jiahuan You, Junxia Wang, Jingwei Cai, Hao Wang, Jie Wang, Wei Zhang, Jicheng Yu, Chunmao Han, Yuqi Zhang, Zhen Gu

**Affiliations:** 1 Key Laboratory for Advanced Drug Delivery Systems of Zhejiang Province, College of Pharmaceutical Sciences, Zhejiang University, 310058 Hangzhou, China; 2 Department of General Surgery, Sir Run Run Shaw Hospital, School of Medicine, Zhejiang University, 310016 Hangzhou, China; 3 Department of Burns and Wound Care Center, the Second Affiliated Hospital, College of Medicine, Zhejiang University, 310009 Hangzhou, China; 4 Jinhua Institute of Zhejiang University, 321299 Jinhua, China; 5 Liangzhu Laboratory, Zhejiang University Medical Center, 311121 Hangzhou, China; 6 National Key Laboratory of Advanced Drug Delivery and Release Systems, Zhejiang University, 310058 Hangzhou, China; 7 MOE Key Laboratory of Macromolecular Synthesis and Functionalization, Department of Polymer Science and Engineering, Zhejiang University, 310027 Hangzhou, China

**Keywords:** Drug delivery, Biomedical materials, Drug delivery

## Abstract

Effective reprogramming of chronic wound healing remains challenging due to the limited drug delivery efficacy hindered by physiological barriers, as well as the inappropriate dosing timing in distinct healing stages. Herein, a core-shell structured microneedle array patch with programmed functions (PF-MNs) is designed to dynamically modulate the wound immune microenvironment according to the varied healing phases. Specifically, PF-MNs combat multidrug-resistant bacterial biofilm at the early stage via generating reactive oxygen species (ROS) under laser irradiation. Subsequently, the ROS-sensitive MN shell gradually degrades to expose the MN core component, which neutralizes various inflammatory factors and promotes the phase transition from inflammation to proliferation. In addition, the released verteporfin inhibits scar formation by blocking *Engrailed-1* (*En1*) activation in fibroblasts. Our experiments demonstrate that PF-MNs promote scarless wound repair in mouse models of both acute and chronic wounds, and inhibit the formation of hypertrophic scar in rabbit ear models.

## Abstract (web summary)

Effective reprogramming of chronic wound healing remains challenging due to the limited drug delivery efficacy hindered by physiological barriers, as well as the inappropriate dosing timing in distinct healing stages. Here, the authors report a core-shell structured microneedle array patch with programmed functions which dynamically modulates the wound immune microenvironment according to the varied healing phases

## Introduction

The classical wound healing process mainly consists of four stages, namely hemostasis, inflammation, proliferation, and remodeling, wherein the regulation of immune microenvironment serves as a key factor to promote the progress in a sequential manner^1, 2^. However, impaired wound healing with dysfunctions of immunoregulation could lead to chronic wounds, which are usually stagnated in the phase of inflammation^1,3^. The delayed wound healing could be ascribed to extensive inflammatory responses primarily driven by bacterial infection, which subsequently cause the failure of macrophage polarization and excessive secretion of pro-inflammatory cytokines including interleukin-1β (IL-1β), IL-6, and tumor necrosis factor-α (TNF-α)^4–6^. Consequently, the imbalance of immune microenvironment impedes the healing process by restraining the formation of granulation tissue, neovascularization, and re-epithelialization^7,8^. Noteworthily, persistent inflammatory response in early stages is directly associated with deficiencies in dermal appendage, leading to insufficient skin functions and subsequent scar formation^1^. Therefore, the specific modulation of wound microenvironment in varied repair phases is tightly demanded in clinics, especially for chronic wounds.

In the last decade, several strategies have been explored for the treatment of chronic wounds by targeting the inflammatory microenvironment around the wound region, such as the delivery of cytokines^9^, the implantation of naturally derived or synthetic scaffolds^10–12^, as well as cell therapy^13,14^. However, the skin barrier system which includes physical, chemical, immunological, and microbiome barriers significantly hinders the transport of active ingredients, thereby limiting the therapeutic efficacy^15^. The presence of bacterial biofilms in wounds can further exacerbate inflammation and restrict the efficacy of antibiotic treatments due to their intrinsic resistance^16^. Although topical antibiotics can help mitigate surface-level infections, they may not be effective against deeper infections because of the limited permeability across the skin barrier. Polymeric microneedles (MNs)^17^, emerging as effective tools for transdermal drug delivery via perforating the stratum corneum, have been utilized for diagnosis and treatment of various diseases including cancer^18^, diabetes mellitus^19^, obesity^20^, and infection^21^. MNs encapsulated with antibiotics or metals have demonstrated superior efficacy in eliminating biofilms over other strategies^22,23^. However, there are still several challenges remaining in MN-mediated wound healing fields^24^. For example, most MNs were designed to inhibit bacterial infection only through the controlled release of encapsulated therapeutics, yet dynamical program of immune microenvironment corresponding to distinct healing stages has not been explored.

**Fig. 1:**
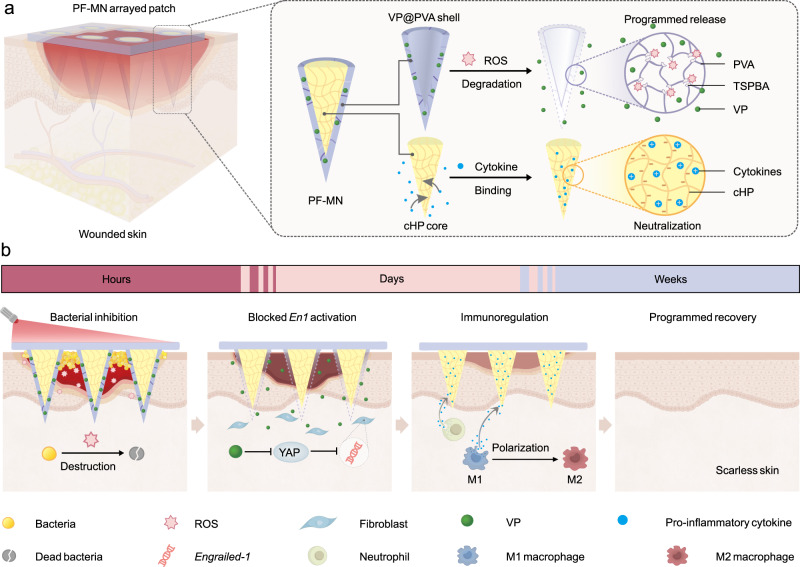
Schematic of scarless wound healing process programmed by core-shell structured PF-MNs. Schematic illustration of **a** the structure of PF-MNs and **b** the programmed regulation process for chronic wound, including elimination of bacterial biofilm via generating ROS, release of VP for scarless wound regeneration, and cytokine neutralization as well as macrophage transformation by cHP core. PVA poly (vinyl alcohol), cHP crosslinked heparin, VP verteporfin, ROS reactive oxygen species, TSPBA a ROS-responsive crosslinker, *En1* *Engrailed-1*, YAP Yes-associated protein.

In this study, we describe a core-shell structured MN patch for the regulation of the inflammation, proliferation, and remodeling phases in a programmed manner (Fig. 1). The MN with programmed functions (PF-MN) was composed of a ROS-degradable poly (vinyl alcohol) shell (VP@PVA shell) loaded with a therapeutic agent, verteporfin (VP), for bacterial inhibition and wound remodeling; and a core component made of crosslinked heparin (cHP core) for immune modulation. Upon application to the wound, the MNs effectively disrupted the bacterial biofilm, and then the embedded VP generated ROS under laser irradiation to eradicate the underneath bacteria, which were hardly managed by antibiotics. Subsequently, the MN shell gradually degraded under oxidative stress along with the development of inflammation stage, resulting in the release of the loaded VP. The released VP, an inhibitor of Yes-associated protein (YAP), could further block *Engrailed-1* (*En1*) activation in fibroblasts to facilitate scarless skin regeneration^25,26^. Meanwhile, the exposed cHP core initiated modulation of immune microenvironment by binding and neutralizing various inflammatory factors to induce macrophage polarization from M1 (pro-inflammatory) to M2 (anti-inflammatory) phenotype, forwarding the phase transition from inflammation to proliferation. In addition, the modulation of immune microenvironment promoted angiogenesis and extracellular matrix deposition, and finally attributed to scarless regeneration. Upon this design, scarless wound healing process was accelerated on both acute wound and diabetic mouse models, as well as on rabbit ear scar models.

## Results

### Fabrication and characterization of the PF-MN patch

**Fig. 2:**
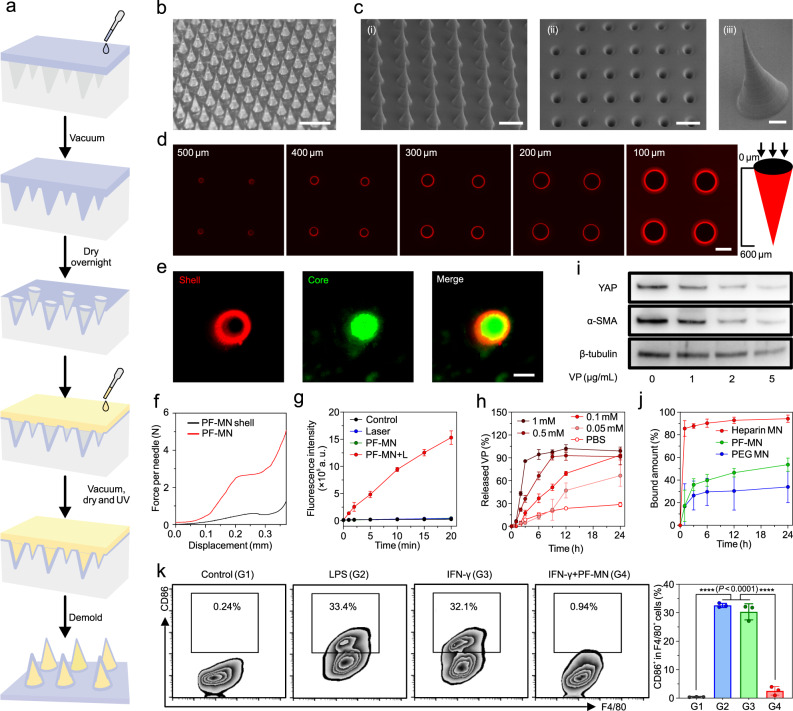
The fabrication process and characterizations of PF-MNs with core-shell structure. **a** Schematic of the fabrication process of PF-MN arrayed patch via mold casting. **b** Representative photograph of the PF-MN patch. Scale bar, 1 mm. **c** The SEM images showing the (i) Side and (ii) Bottom view of the shell of PF-MN patch. Scale bar, 500 μm. (iii) The enlarged SEM image displaying one PF-MN. Scale bar, 100 μm. **d** Representative confocal images of Rhodamine B labeled PF-MN shell from a bottom view. The intervals at z-direction were set as 100 μm. Scale bar, 200 μm. **e** Representative images of the core-shell structured PF-MN. DiI-labeled PVA shell (red) and FITC-labeled heparin core (green). Scale bar, 100 μm. **f** Mechanical strength of MN shell and PF-MN. **g** ROS generation from PF-MNs loaded with VP (3 μg) under 690 nm laser irradiation (25 mW/cm^2^) (*n* = 3 independent samples). **h** Accumulated release of VP from PF-MN shell in PBS with different concentrations of H_2_O_2_ (*n* = 3 independent samples). **i** Protein levels of YAP and α-SMA in fibroblasts treated with different concentrations of VP as determined by Western blot. **j** Binding kinetics of IFN-γ by differently charged MNs (*n* = 4 independent samples). **k** Flow cytometry analysis indicating the transformation of macrophages towards M1 phenotype induced by IFN-γ could be inhibited by PF-MNs (*n* = 3 independent samples). Three independent experiments were performed and representative results are shown in **c**–**e**. Data are presented as mean ± SD and statistical significance was analyzed via one-way ANOVA with Tukey’s multiple comparison test. *P* value: ****P < 0.0001.

To engineer the core-shell structured PF-MN patch, the degradable MN shell was first prepared with poly (vinyl alcohol) (PVA, 2%) and a ROS-sensitive linker *N*^1^-(4-boronobenzyl)-*N*^3^-(4-boronophenyl)-*N*^1^,*N*^1^,*N*^3^,*N*^3^-tetramethylpropane-1,3-diaminium (TSPBA, 2%) at a volume ratio of 2:1 (Supplementary Fig. 1)^27^. Then, photo-crosslinked heparin formed the core of MN (Fig. 2a and Supplementary Fig. 2). VP was further loaded to the above blank PF-MNs. A representative PF-MN patch was arranged in a 32 × 32 array with conical needles of 600 μm in height and 300 μm in base diameter (Fig. 2b). The hollow structure of the MN shell was observed using the scanning electron microscope (SEM; Fig. 2c) and confocal microscope (Fig. 2d and Supplementary Fig. 3). The core-shell structure was further confirmed by the cross-section view of PF-MNs (Fig. 2e). The mechanical strength of PF-MN was determined as around 2.7 N per needle, which was sufficient to penetrate wounds^28^, while the strength of PF-MN shell was only 0.5 N per needle (Fig. 2f).

Next, we explored the antibacterial potential of PF-MNs for the treatment of bacterial-infected wounds. The loaded VP is a second-generation photosensitizer in the family of benzoporphyrins, which is approved by FDA for the photodynamic therapy (PDT) of macular degeneration clinically^29^. Sustained ROS generation by VP under laser irradiation was demonstrated as the increased fluorescence signal of 2′,7′-dichlorodihydrofluorescein diacetate (DCFH-DA; Fig. 2g). In addition, the structure of VP did not change after laser irradiation for 10 min, indicating its desired stability (Supplementary Fig. 4).

The immune-regulative performance of PF-MNs was also assessed in vitro. Pro-inflammatory macrophage and cytokines are known to evoke host defense and promote phagocytosis, playing crucial roles in the early response to injury^30^. However, constant presence of pro-inflammatory macrophage could lead to chronic inflammation. In order to promote phase transition from inflammation to proliferation, the cHP was designed to be sheathed by the degradable MN shell during the earlier period of the inflammation stage (first early 24 h), but latterly exposed to neutralize pro-inflammatory cytokines^31,32^. To investigate the degradation behavior of the VP@PVA shell, 0.1 mM H_2_O_2_ was selected to simulate the inflammatory microenvironment of chronic wounds^33^. The release profiles of VP from shells with different ratios of PVA and TSPBA were investigated (Supplementary Fig. 5), and the ratio of 2:1 was determined considering its appropriate degradation duration (~24 h). Moreover, the release rate of VP from PF-MNs could also be tuned by the concentration of H_2_O_2_ (Fig. 2h). Recent reports indicated that VP could block *Engrailed-1* (*En1*) activation by inhibiting Yes-associated protein (YAP), yielding skin regeneration with less alpha-smooth muscle actin (α-SMA), a marker of activated myofibroblasts that contributed to scar formation^25,26^. Western blot analysis confirmed these down-regulations of YAP and α-SMA protein expression in fibroblasts treated with VP (Fig. 2i).

After the degradation of the MN shell, the cHP core was exposed to the inflammatory microenvironment for neutralizing pro-inflammatory cytokines. The binding kinetics of the cHP core to inflammatory cytokines such as interferon-γ (IFN-γ) was determined by incubating differently charged MNs with IFN-γ. Heparin-formulated MNs (negatively charged) could bind with IFN-γ to reach over 90% neutralization. In contrast, only less than 30% of IFN-γ was neutralized by polyethylene glycol (PEG)-formulated MNs (neutrally charged). The crosslinked PVA shell was also validated to isolate the inner heparin core from the outer environment with limited binding of IFN-γ (Fig. 2j). Similarly, heparin-formulated MNs could also efficiently adsorb inflammatory cytokines and chemokines including tumor necrosis factor-α (TNF-α) and monocyte chemoattractant protein-1 (MCP-1) (Supplementary Fig. 6). In addition, the cHP core showed promising stability with only slight dissociation for 96 h (Supplementary Fig. 7). The consumption of chemokines from the inflamed wound could impede the recruitment of immune cells and restrain the polarization of macrophage towards pro-inflammatory phenotype^31^. Lipopolysaccharide (LPS) and IFN-γ were served as positive control groups to promote the M1 phenotype transition of RAW 264.7 cells. In contrast, the polarization of macrophage was significantly restricted by neutralizing IFN-γ after the treatment of PF-MNs (Fig. 2k).

### In vitro antibacterial and anti-biofilm activities of PF-MNs

**Fig. 3:**
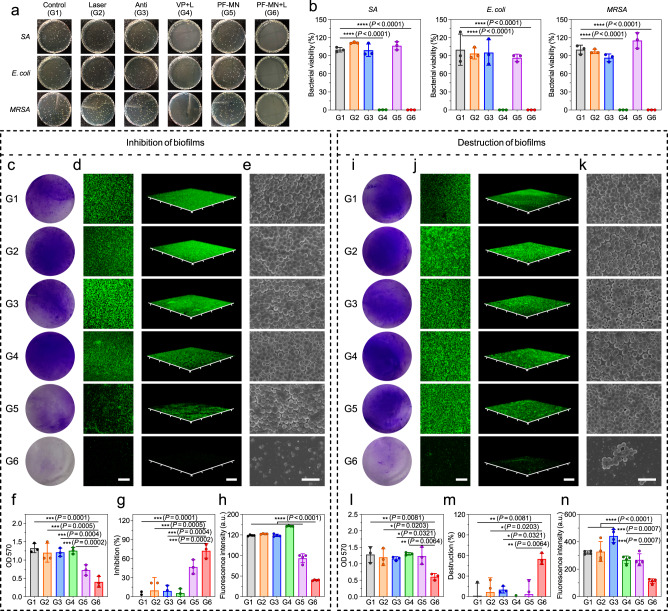
Evaluation of antibacterial and anti-biofilm activities by PF-MNs. **a** Photographs of bacterial colonies of *SA*, *E. coli*, and *MRSA* on LB agar plates after different treatments. **b** Corresponding bacterial viability of *SA*, *E. coli*, and *MRSA* after different treatments (*n* = 3 independent samples). **c** Photographs of the immature biofilms stained by crystal violet, showing the inhibitory effects of PF-MNs. **d** Confocal images and corresponding 3D images of DMAO-stained (green) immature biofilms after different treatments. Scale bar, 200 μm. **e** The SEM images of immature biofilms with different treatments after the gradient dehydration. Scale bar, 3 μm. **f**, **g** Corresponding OD 570 value (**f**) and inhibition rate (**g**) of immature biofilms of each group (*n* = 3 independent samples). **h** Corresponding fluorescence intensities of DMAO-stained immature biofilms (*n* = 4 independent samples). **i** Photographs of the mature biofilms stained by crystal violet, showing the destructive effects of PF-MNs. **j** Confocal images and corresponding 3D images of DMAO-stained (green) mature biofilms after different treatments. Scale bar, 200 μm. **k** The representative SEM images with different treatments after the gradient dehydration of mature biofilms. Scale bar, 3 μm. **l**–**m** Corresponding OD 570 value (**l**) and destruction rate (**m**) of mature biofilms (*n* = 3 independent samples). **n** Corresponding fluorescence intensities of DMAO-stained mature biofilms (*n* = 4 independent samples). L Laser. Three independent experiments were performed and representative results are shown in **c**–**e**, **i**–**k**. Data are presented as mean ± SD and statistical significance was analyzed via one-way ANOVA with Tukey’s multiple comparison test. *P* value: *P < 0.05, **P < 0.01, ***P < 0.001, ****P < 0.0001.

We evaluated the antibacterial performance of PF-MNs based on bacterial colony counting^34^. PF-MNs with laser irradiation (PF-MN + L group) showed nearly 100% inhibition towards Gram-positive *Staphylococcus aureus* (*SA*), Gram-negative *Escherichia coli* (*E. coli*), *Pseudomonas aeruginosa* (*PA*), and Methicillin-resistant *Staphylococcus aureus* (*MRSA*), indicating its broad and efficient antibacterial property (Fig. 3a, b and Supplementary Fig. 8a, b). On the contrary, PF-MNs without laser irradiation (PF-MN group) and laser alone group showed limited effect against bacteria.

A more challenging fate is the formation of bacterial biofilms in chronic wounds. As a self-synthesized matrix of hydrated extracellular polymeric substances, bacterial biofilm is difficult to be eradicated by traditional treatment of antibiotics, especially those formed by drug-resistant bacteria such as *MRSA*^15^. MNs were considered as a promising system to disrupt formed biofilms and deliver antibacterial agents underneath^22^. As depicted in anti-biofilm experiments, the treatment of PF-MNs with laser irradiation showed higher inhibitive efficacy than antibiotics towards both immature biofilms formed by the antibiotic-sensitive *PA* and the drug-resistant *MRSA* (Supplementary Fig. 8c-f and Fig. 3c–h). An ideal inhibition rate of the PF-MN + L group (72.39%) towards the immature *MRSA* biofilms was achieved in the PF-MN + L group. Besides the immature biofilms, we also demonstrated that PF-MNs could disrupt mature *PA* and *MRSA* biofilms (Supplementary Fig. 8g-j and Fig. 3i–n). On the contrary, the treatment with VP solution under laser irradiation showed limited inhibitive effects on biofilms (<10%), demonstrating the indispensable role of MNs on the destruction of biofilms.

### Promoted wound healing by PF-MNs via modulating immune microenvironment

Encouraged by the in vitro results, we further evaluated the ability of PF-MNs to propel wound healing process in an acute postsurgical wound model. Full-thickness wounds were established on the back of healthy BALB/c mice by perforating the skin (Supplementary Fig. 9a). The mice were divided into four groups: (1) Control group (PBS), (2) VP group (VP solution), (3) blank PF-MN group (blank PF-MNs without VP), and (4) PF-MN group (VP-loaded PF-MNs). As shown in Supplementary Fig. 9b, c, the introduction of blank PF-MNs and VP-loaded PF-MNs both led to expedited wound closure on day 7. On day 14, the wounds treated with PF-MNs reached almost complete healing with smooth and neonatal skin.

We hypothesized that the accelerated wound healing was attributed to the immune-regulatory capacity of the cHP core in the PF-MNs. Therefore, the amounts of cytokines in the wound tissue were analyzed by enzyme-linked immunosorbent assay (ELISA). A variety of pro-inflammatory cytokines including IFN-γ, IL-6, and MCP-1 were decreased after both blank PF-MN and VP-loaded PF-MN treatments (Supplementary Fig. 9d–f). We also assessed the infiltration of immune cells in the wounds by flow cytometry analysis (Supplementary Fig. 10). Macrophages were recognized for playing crucial roles in host defense and immune regulation via polarizing to various phenotypes^7^. Ineffective phenotypic transition (M1 to M2) often leads to chronic or unhealable wounds^1^. Thus, we explored the polarization of macrophages around the wounds on day 3 post-treatments through flow cytometry. We found that the administration of PF-MNs significantly reduced the proportion of M1 phenotype (F4/80^+^CD86^+^) macrophages. Moreover, the percentage of M2 phenotype (F4/80^+^CD206^+^) macrophages showed an elevation in the blank PF-MN group (16.12%) and PF-MN group (23.52%) compared to the Control group (8.46%) and VP group (13.37%), indicating that PF-MNs could promote the polarization of macrophage towards anti-inflammatory phenotype in the presence of cHP core. Besides macrophages, regulatory T cells (Tregs) also make important contributions to the regulation of immune responses (Supplementary Fig. 9g, h)^35^. The proportions of Tregs (CD4^+^FoxP3^+^) in the PF-MNs involved groups were enhanced, which was favorable for wound recovery. Moreover, haematoxylin and eosin (H&E) staining also showed that tightly connected epidermis was formed in the blank PF-MN group and PF-MN group on day 7 (Supplementary Fig. 9i, j). While more smooth skin surface with unnoticeable scar formation was observed in the PF-MN group, which could be attributed to the presence of VP.

### Alleviated chronic inflammation and promoted wound regeneration by PF-MNs in diabetic wounds

We further evaluated whether PF-MNs could promote chronic wound regeneration. Diabetic foot ulcer (DFU), one of the major complications in diabetic people, is hard to heal spontaneously due to its vulnerability to bacterial infection, prolonged inflammation, impaired angiogenesis, and limited cell proliferation^36,37^. The hyperglycemic and hostile environment surrounding the defect area often provides a suitable ground for bacterial colonization. When a biofilm forms, especially one induced by multidrug-resistant bacteria, conventional treatments will no longer be effective^38^.

**Fig. 4:**
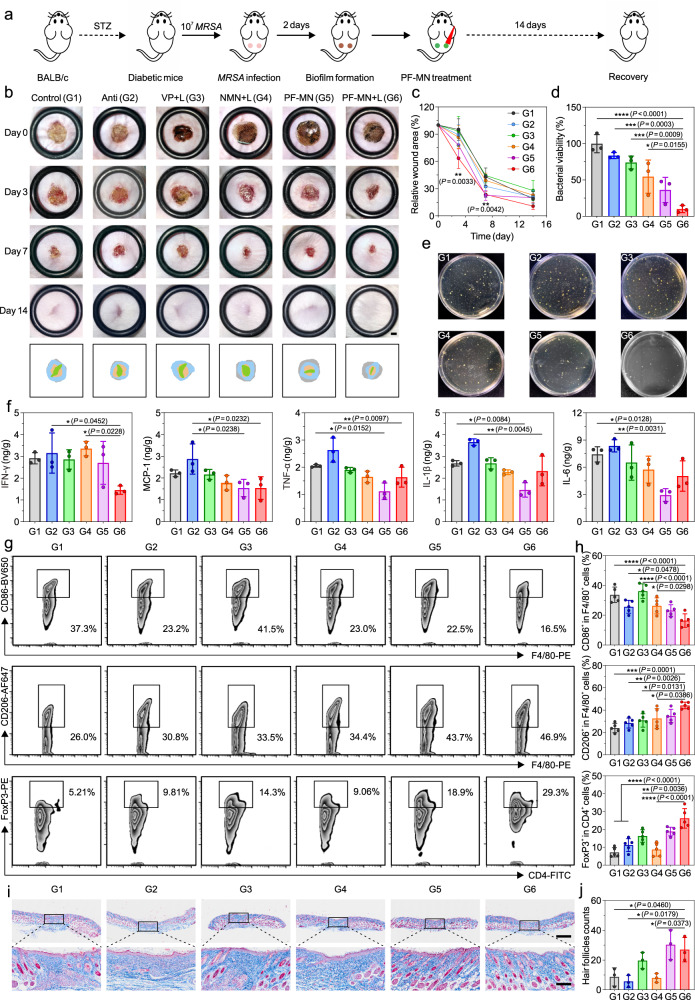
In vivo wound healing efficacy on diabetic mice infected by *MRSA*. **a** Experimental illustration presenting PF-MN-mediated wound healing on diabetic mice infected with *MRSA*. **b** Photographs of wounds of BALB/c mice at different time points after varied treatments. Scale bar, 2 mm. **c** Quantitative analysis of the relative wound areas at different times (*n* = 6 biologically independent samples). **d**, **e** Bacterial viability (**d**) and corresponding photographs (**e**) of bacterial colonies of *MRSA* on LB agar plates in the wounds on day 3 post-treatments (*n* = 3 biologically independent samples). **f** Quantitative analysis of the levels of IFN-γ, MCP-1, TNF-α, IL-1β, and IL-6 from the wounds on day 3 post-treatments (*n* = 3 biologically independent samples). **g**, **h** Representative flow cytometry plots (**g**) of macrophage and regulatory T cell analyzed from wounds on day 3 and corresponding quantitative results (**h**) (*n* = 5 biologically independent samples). **i**, **j** Representative Masson Trichrome staining images (**i**) of wounds on day 14 and quantitative analysis of hair follicles (**j**). Scale bar, 1 mm (top) and 200 μm (enlarged) (*n* = 3 biologically independent samples). Three independent experiments were performed and representative results are shown in **i**. Data are presented as mean ± SD and statistical significance was analyzed via one-way ANOVA with Tukey’s multiple comparison test. *P* value: *P < 0.05, **P < 0.01, ***P < 0.001, **** =*P* < 0.0001.

In the chronic wound model, *MRSA* biofilms were generated two days post the introduction of *MRSA* into the wounds created on the back of streptozotocin (STZ)-induced diabetic mice (Fig. 4a)^34,39^. The mice were divided into six groups randomly: (1) Control group (PBS), (2) Anti group (ampicillin), (3) VP + L group (VP solution with laser irradiation), (4) NMN + L group (VP-loaded non-degradable MNs with laser irradiation), (5) PF-MN group (VP-loaded PF-MNs), and (6) PF-MN + L group (VP-loaded PF-MNs with laser irradiation). The wound areas in different groups were measured and photographed on day 0, 3, 7, and 14. As shown in Fig. 4b, c, the wounds treated with PF-MNs exhibited accelerated wound closure. The wound healing process with laser irradiation was further expedited. Quantitatively, the PF-MN + L group led to 36.58% and 76.46% wound closure on day 3 and 7 post-treatment respectively, which were dramatically higher than the PBS-treated wounds (4.89% and 56.95%, respectively). By collecting and homogenizing the skin tissue around wound areas on day 3, the anti-biofilm performance was evaluated (Fig. 4d, e). The wounds treated by PF-MNs with laser irradiation showed the lowest number of bacterial colonies with a bacterial viability rate of 18.60%, indicating effective inhibition of bacterial biofilms.

We also found the levels of various pro-inflammatory cytokines mainly released by neutrophils and M1 macrophages, such as IFN-γ, MCP-1, TNF-α, IL-1β, and IL-6, were down-regulated in the PF-MN group and PF-MN + L group (Fig. 4f), further validating that cHP core could neutralize the inflammatory environment by flow cytometry analysis (Supplementary Fig. 11). The results showed that the percentages of M1 phenotype macrophage in the PF-MN involved groups were lower than the PBS-treated group. Treatment of PF-MNs with laser irradiation further reduced the proportion of M1 phenotype macrophages by eradicating bacteria. In contrast, the proportions of M2 phenotype macrophages and Tregs in the PF-MN involved groups were found to be elevated, which benefited the wound recovery (Fig. 4g, h). Moreover, various immune cells including lymphocytes, monocytes, neutrophils, and eosinophils decreased with the treatment of PF-MNs as indicated by the blood routine analysis (Supplementary Fig. 12).

The immunofluorescence staining showed the total amounts of macrophage in the wounds with different treatments almost remained the same (Supplementary Fig. 13a, b). However, the levels of CD86 (M1 phenotype macrophage) in the groups treated by PF-MNs with/without laser irradiation were significantly lower than the PBS-treated group (Supplementary Fig. 13c). On the contrary, the level of CD206 (M2 phenotype macrophage) increased by five times with the involvement of PF-MNs (Supplementary Fig. 13d). These results were consistent with the above flow cytometry analysis, evidencing that PF-MN treatment promoted the polarization of macrophage towards anti-inflammatory phenotype in chronic wounds. The level of platelet endothelial cell adhesion molecule-1 (CD31) in the PF-MN + L group was about two-fold higher than that in the PBS-treated group on day 7, indicating facilitated angiogenesis by programmed modulation of wound microenvironment (Supplementary Fig. 13e). In addition, α-SMA immunofluorescent staining indicated that the delivery of VP by PF-MNs could down-regulate the expression of α-SMA in fibroblasts, thus inhibiting scar formation during the remodeling stage (Supplementary Fig. 13f).

More detailed wound healing processes were investigated by histological analysis, including H&E staining, Masson Trichrome staining, and Sirius Red staining^40^. The wounds in the PBS-treated group exhibited apparent inflammation as indicated by the infiltration of immune cells (H&E staining image, day 3), while the inflammation was relieved after the treatment of PF-MNs with laser irradiation (Supplementary Fig. 14a). Subsequently, the skin treated with PF-MNs presented more consecutive epithelial tissues and well-defined granulation tissues on day 7 compared with other groups. The analysis of the epidermal thickness also indicated the satisfactory repair of epidermal tissue on day 7 (Supplementary Fig. 14b). Two weeks post-treatment, a scarless mature skin with more hair follicles was observed in the wound treated with PF-MNs.

Masson Trichrome staining also revealed a defined arrangement and higher volume fraction of collagen in the PF-MNs involved groups, confirming their ability to facilitate the deposition of extracellular matrix (Fig. 4i, j). Moreover, Sirius Red staining was conducted to explore whether the introduction of PF-MNs could prevent the formation of scar. Type I collagen is usually considered as the prominent part of the extracellular matrix of hypertrophic scar (HS), and the ratio of type I/III collagen in HS is usually higher than that in normal skin^41^. As shown in Supplementary Fig. 14c, the ratios of type I (red) to type III (green) collagen in both groups treated by PF-MNs with and without laser irradiation were much lower than the PBS-treated group, revealing the scar mitigation effect of PF-MNs. In addition, PF-MNs also performed desired biosafety as evidenced by the biochemistry analysis (Supplementary Fig. 15). H&E staining images of major organs (heart, liver, spleen, lung, and kidney) from diabetic mice on day 14 showed insignificantly morphological and structural changes compared with the PBS-treated mice (Supplementary Fig. 16).

We further conducted the transcriptomic analysis to investigate the potential mechanism of accelerated wound healing by PF-MNs. The results indicated significant differences in gene expression among the groups (PF-MN + L versus NMN + L and Control), with 218 up-regulated and 509 down-regulated genes shown in the volcano plots (Supplementary Fig. 17a). The results in the heatmap revealed that genes related to immune response were down-regulated in the group treated with PF-MNs and laser irradiation, such as Toll like receptor 1 (TLR1), C–C motif chemokine ligand 2 (CCL2) and C-X-C motif chemokine ligand 2 (CXCL2) (Supplementary Fig. 17b). The differentially expressed genes were collected to perform Gene Ontology (GO) database analysis, including biological process (BP), cellular component (CC), and molecular function (MF). The genes associated with wound repair including epidermis development and skin development were up-regulated in the PF-MN + L group, while the terms related to the pathways of inflammation were down-regulated, such as cytokine-mediated signaling, leukocyte chemotaxis, IL-6 production, and regulation of inflammatory response (Supplementary Fig. 17c, d). Furthermore, the potential signaling pathways were analyzed with the Kyoto Encyclopedia of Genes and Genomes (KEGG). As depicted in Supplementary Fig. 17e, peroxisome proliferator-activated receptor (PPAR) signaling pathway was up-regulated, which was associated with M2 macrophage activation. By contrast, TNF and nucleotide-binding oligomerization domain (NOD)-like receptor signaling pathways were down-regulated as shown in Supplementary Fig. 17f, which were considered as M1 activation-related pathways^42^. Collectively, the results validated that the PF-MNs accelerated chronic wound healing through suppressing pro-inflammatory pathways and promoting skin reconstructive pathways.

### Scarless wound healing programmed by PF-MNs

**Fig. 5:**
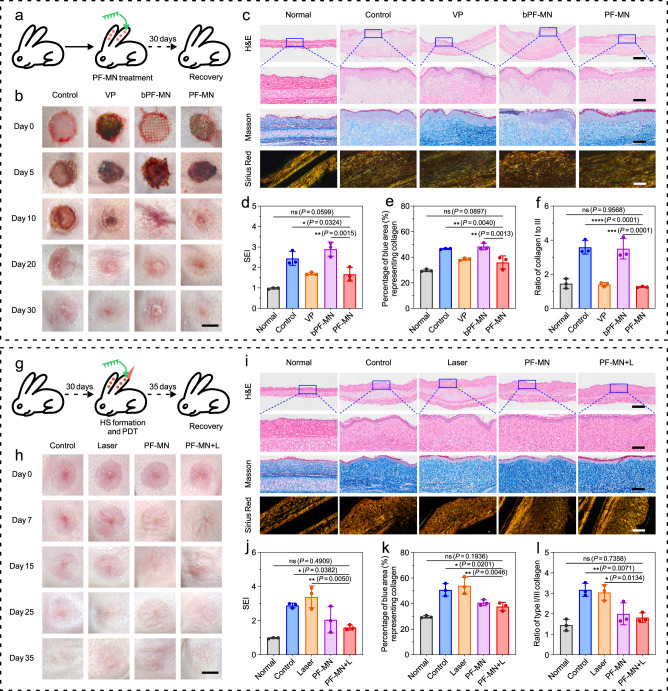
Inhibition of scar formation and alleviation of HS by PF-MNs on rabbit ears. **a** Experimental schematic of PF-MN assisted scarless wound healing on rabbit ears. **b** Photographs of wounds on rabbit ears with varied treatments at different time points. Scale bar, 5 mm. **c** Representative H&E staining, Masson Trichrome staining, and Sirius Red staining images of wounds on day 30. Scale bar, 1 mm in H&E staining images (top), 200 μm in H&E staining images (enlarged), Masson Trichrome staining, and Sirius Red staining images. **d**–**f** Quantitative analysis of SEI (**d**), percentage of collagen (**e**), and type I/III collagen ratio (**f**) of wounds (*n* = 3 biologically independent samples). **g** Experimental schematic of PF-MNs for PDT of HS. **h** Photographs of scars on rabbit ears after varied treatments at different times. Scale bar, 5 mm. **i** Representative H&E staining, Masson Trichrome staining, and Sirius Red staining images of HS on day 15. Scale bar, 1 mm in H&E staining images (top), 200 μm in H&E staining images (enlarged), Masson Trichrome staining, and Sirius Red staining images. **j**–**l** Quantitative analysis of SEI (**j**), percentage of collagen (**k**), and type I/III collagen ratio (**l**) of HS (*n* = 3 biologically independent samples). bPF-MN: blank PF-MN. Three independent experiments were performed and representative results are shown in **c** and **i**. Data are presented as mean ± SD and statistical significance was analyzed via one-way ANOVA with Tukey’s multiple comparison test. *P* value: *P < 0.05, **P < 0.01, ***P < 0.001, ****P < 0.0001.

We then assessed the capability of scar inhibition via deep delivery of VP by MNs in a rabbit HS model (Fig. 5a), which is more similar to human beings^43,44^. The rabbits were divided into four groups randomly: (1) Control group (PBS), (2) VP group (VP solution), (3) blank PF-MN group (blank PF-MNs without VP), and (4) PF-MN group (VP-loaded PF-MNs). As shown in Fig. 5b, both blank PF-MN and PF-MN treated groups showed accelerated wound healing. However, HS was formed on day 30 with red color and raised scar surface in the PBS-treated group and blank PF-MN-treated group. Instead, more flattened HS tissue was observed after the treatment of VP solution and VP-loaded PF-MNs, indicating the vital function of VP to reduce HS formation. From the histological analysis, we observed the hypertrophic features in the PBS-treated wounds as well (Fig. 5c). In contrast, the introduction of VP (VP group and PF-MN group) contributed to thinner and flatter epidermis, which was further evidenced by the scar evaluation index (SEI) (Fig. 5d)^41,43,45^. Masson Trichrome staining and Sirius Red staining results also revealed that the collagen fibers (blue) in skin treated with VP solution and PF-MNs were significantly reduced compared to that in other two groups (Fig. 5e). Additionally, the ratio of type I/III collagen dropped from 3.59 (Control group) to 1.40 (VP group) and 1.26 (PF-MN group) respectively, indicating that both free VP and embedded VP could mitigate scar formation (Fig. 5f).

### Transdermal PDT on hypertrophic scar by PF-MNs

PDT is able to trigger cell apoptosis and decrease collagen deposition by generated ROS (Supplementary Fig. 18), thus possessing potentials for recovering HS^44, 46^. To assess the feasibility of PF-MNs to treat already formed scar, the HS tissue with raised red surfaces were formed by re-epithelialization of the wound on day 30 after skin resection^41^. By introducing PF-MNs accompanied with laser irradiation, the HS tissue became softened and flattened (Fig. 5g, h). On the contrary, the other three groups still exhibited pathological appearances with firm texture and contracted tissues.

H&E staining indicated that the untreated HS displayed infiltration of proliferative fibroblasts, along with prominent hyperplasia in the epidermis and dermis (Fig. 5i). Those scar symptoms could not be significantly alleviated by laser irradiation or the treatment of PF-MNs. In contrast, the combination of PF-MNs and laser irradiation led to considerable therapeutic efficacy with the thinner dermis and flatter epidermis, even close to the normal level. As shown in Fig. 5j, the SEI value of this group also exhibited a relative decrease compared to the PBS-treated group on day 15. Furthermore, there was reduced deposition of collagen and decreased ratio of type I/III collagen within the tissue after the combinational treatment of PF-MNs and laser according to the Masson Trichrome staining (Fig. 5k, l). Similar trends were also observed on day 25 and 35, as shown in Supplementary Fig. 19.

## Discussion

Chronic wound healing poses a significant challenge to the global healthcare system. Prolonged inflammation is believed to be responsible for stalled or impaired wound healing^5^. Advancing the repair progress to a normal well-orchestrated manner may contribute to the desired wound healing. However, bacterial infiltration stands out as a major impediment to wound closure, particularly in people with diabetes. Conventional antibiotics often fail to eliminate biofilms produced by drug-resisted bacteria, such as *MRSA*, resulting in persistent inflammatory response and delayed healing process^38^. Although PDT has emerged as a promising strategy for treating bacterial infections by generating highly toxic ROS recently, their antibacterial efficiency is restrained by the limited penetration through biofilms. Relying on the physical penetrating ability of MNs to disrupt mature bacterial biofilms, the developed PF-MNs could generate ROS underneath to effectively inhibit the bacterial infection by the embedded photosensitizer VP.

Besides bacterial infection, macrophages play an important role in regulating inflammatory response^4,7^. The polarization of macrophages from M1 (pro-inflammatory) to M2 (anti-inflammatory) and maintaining appropriate levels of inflammatory factors are essential for forwarding the wound healing stages from inflammation to regeneration. Glycosaminoglycan has been reported to neutralize various inflammatory cytokines^31^. In this work, heparin was chosen to formulate the core of MN for binding and neutralizing inflammatory cytokines, such as IFN-γ, MCP-1, TNF-α, IL-1β, and IL-6. Furthermore, the inhibition of bacteria and depletion of pro-inflammatory factors also contributed to the polarization of macrophages from M1 phenotype to M2 phenotype, thus remarkably accelerating wound regeneration.

During the remodeling stage, excessive scar formation represents another major obstacle in skin regeneration, resulting from excessive collagen synthesis, deficient matrix degradation, and failed coordination of various cells, especially under pathological conditions^1^. In addition, persistent inflammation and dysfunction of immunoregulation are also associated with scar formation^1,7,26^. Recent studies revealed *Engrailed-1* lineage-positive fibroblasts (EPFs) were crucial in aggravating scar formation, and suppression of *En1* activation by inhibiting YAP using VP could facilitate scarless skin regeneration^25^. Effective delivery of VP to the deeper tissues by transdermal MNs in our study was able to remold the ultrastructure of the scar tissue. The remarkable decrease in the thickness of scar, the proportion of collagen, and the ratio of type I/III collagen were observed in the wounds treated by this approach.

In summary, we developed a core-shell structured PF-MN patch to advance the wound healing process in a programmed manner. In the early stage of inflammation, PF-MNs generated ROS under laser irradiation for bactericidal effects. Afterwards, the VP@PVA shell responsively degraded upon the highly oxidative wound environment and released VP to promote scarless skin regeneration. Meanwhile, the exposed cHP core modulated immune microenvironment via neutralizing excessive inflammatory cytokines and inducing macrophage polarization. Our findings suggested that this core-shell structured PF-MN patch could promote chronic wound healing in the *MRSA*-infected diabetic mice, and also attenuate HS in rabbit ear scar models. For potential human use, the PF-MNs could be readily tailed to fit the size of wound and applied onto the wound area. After a short administration of laser irradiation, the PF-MNs allow the wound to experience a self-regulated healing process without any change of the dressing for patients. In addition, the degradation rate of the shell and the release profile of VP could be further dynamically tuned according to the inflammatory condition in the wounds, potentiating personalized treatment in the clinic. Overall, our work presents a strategy to deal with non-healing wounds via core-shell structured MNs, and highlights the significance of programmed modulation of the repair process.

## Methods

### Materials

Heparin sodium (Mw 15 k-19 kDa), verteporfin (97%, VP), 4-(bromomethyl) phenylboronic acid (98%), MES (≥99.5%), ampicillin, streptozotocin (98%, STZ), and crystal violet (98%) were purchased from Macklin. Poly (vinyl alcohol) (Mw ~75 kDa, 92.0–94.0% hydrolysis, PVA), poly (vinyl alcohol) (Mw ~47 kDa, 99% hydrolysis), *N*-hydroxysuccinimide (98%, NHS), 1-(3-dimethylaminopropyl)-*N*′-ethylcarbodiimide hydrochloride (98%, EDC), 2-hydroxy-4′-(2-hydroxyethoxy)−2-methylproplophenone (≥98.0%), ethylene glycol dimethacrylate (98%), and tween 20 were purchased from Aladdin. *N*,*N*,*N*′,*N*′-tetramethyl-1,3-propanediamine (≥99%), 2-aminoethyl methacrylate hydrochloride (90%, AEMA), and poly (ethylene glycol) diacrylate (PEG) were purchased from Sigma-Aldrich. 2′,7′*-*Dichlorodihydrofluorescein diacetate (DCFH-DA) was purchased from Med Chem Express USA. Live & dead bacterial staining kit and Luria-Bertani (LB) medium were purchased from Yeasen Biotech. LB Agar (CM159) was purchased from Beijing land bridge technology. Cell Counting Kit-8 (CCK-8) was purchased from Beijing Lablead Biotech.

### Synthesis of TSPBA

4-(Bromomethyl) phenylboronic acid (1.5 g, 7.0 mmol) was dissolved in dimethyl formamide (DMF) (60 mL) at 60 °C. *N*,*N*,*N*′,*N*′-tetramethyl-1,3-propanediamine (0.15 g, 1.2 mmol) was added into the mixture and stirred for 24 h. Then, the light yellow solution was precipitated in tetrahydrofuran (THF) (150 mL) and filtrated quickly. The resulting white solid was further washed with THF for three times to remove the excess DMF. Pure TSPBA was obtained after volatilizing THF under vacuum overnight^27,47^. The structure was characterized by ^1^H NMR (BRUKER AVIII500M).

^1^H NMR (500 MHz, D_2_O): δ(ppm) = 7.77 (d, 4H), 7.47 (d, 4H), 4.49 (s, 4H), 3.30 (m, 4H), 3.02 (s, 12H), 2.34 (m, 2H).

### Synthesis of m-heparin

Heparin (1 g) was dissolved in MES buffer (50 mM, pH 6.5, NaCl 0.5 M, 100 mL) under stirring. Then, NHS (203 mg) and EDC (676 mg) were added into the solution to activate carboxyl groups of heparin. After reacting for 1 h, AEMA (438 mg) was further added to the above solution. The mixture was stirred at room temperature for 24 h. Then, the solution was precipitated with acetone, and the supernatant was discarded. The white solid was dried in a fume hood, and then hydrated to 1% with deionized (DI) water. After dialysis for three days, m-heparin was obtained with the method of lyophilization and characterized by ^1^H NMR^48^.

^1^H NMR (500 MHz, D_2_O): δ(ppm) = 6.13 (s, 1H), 6.09 (s, 1H), 1.98 (d, 3H).

### Fabrication of PF-MN patch

To fabricate the PF-MN patch with a core-shell structure, PVA (92.0-94.0% hydrolysis, 2%) and TSPBA (2%) were first dissolved in DI water, and then mixed at the volume ratio of 2:1. 2 mL of the mixture was casted into the PDMS mold. The PDMS mold was placed under vacuum to allow the mixture to fill down to the MN tip. Then, the crosslinked PVA, the shell of PF-MN patch, was obtained after air-dried overnight at room temperature. m-heparin (0.1 g), ethylene glycol dimethacrylate (1.5%, 1.5 μL) and photoinitiator (Irgacure 2959, 2%, 2 mg) dissolved in DI water (0.5 mL) were added to the above of hollow MN patch and then filled up the cavity under vacuum. After drying at room temperature in the dark overnight, the blank PF-MN patch was obtained under UV exposure for 20 min. The excessive heparin served as the backing of MN patch and then 30 μg VP was further loaded to the above MNs to obtain PF-MNs. The non-degradable MN patch was obtained with PVA (99% hydrolysis) using the same method. The morphology of PF-MNs was obtained by the scanning electron microscope (SEM, Nova Nano 450) and confocal laser scanning microscopy (CLSM, Olympus FV1000).

### In vitro release of VP

To evaluate the ROS-responsive behavior of PF-MNs, the PF-MN shells loaded with 150 μg VP with different ratios of PVA:TSPBA (1:1, 2:1, 3:1, and 4:1) were incubated in 0.1 mM H_2_O_2_ solutions (5 mL) at room temperature on an orbital shaker (200 rpm), respectively. At predetermined time points, 200 μL of the supernatant was taken out to 96-well plates for analysis. Absorbance at 689 nm was read out with the Hybrid Multi-Mode Reader (Synergy H1, Biotek) to measure the release profiles of VP. The release profiles of VP from MN shells of the ratio of 2:1 in H_2_O_2_ solutions with different concentrations (0 mM, 0.05 mM, 0.1 mM, 0.5 mM, and 1 mM) were measured using the same method.

### Evaluation of ROS generation in vitro

DCFH-DA was used as the ROS indicator. First, sodium hydroxide was used to convert DCFH-DA to DCFH, and then PF-MNs loaded with VP (3 μg) were added into DCFH solution (5 μM in PBS, 500 μL) in a 48-well plate^12^. The PF-MN patch was irradiated under 690 nm laser (25 mW/cm^2^). The fluorescence intensity (488 nm/525 nm) of DCFH was continuously monitored with Hybrid Multi-Mode Reader at predetermined time points. Furthermore, 10^4^ L929 cells were cultured in the complete cell media of dulbecco’s modified Eagle’s medium (DMEM, Gibco) containing 10% fetal bovine serum (FBS, Sigma) and 1% penicillin-streptomycin liquid (100×, Solarbio) in a 24-well plate and incubated at 37 °C for 24 h. VP (5 μg/mL) was added to incubate with cells for 4 h. DCFH-DA (5 μM) was added and incubated for 20 min. Afterwards, VP in the cells was irradiated with 690 nm laser (25 mW/cm^2^) for 10 min to generate ROS and further observed with a fluorescent microscope (Nikon).

### Western bolt analysis

L929 fibroblasts were cultured in a 12-well plate for 24 h. Then, different concentrations of VP were added to the wells for another 24 h. 200 μL of RIPA lysis buffer (Beyotime) was added to each well for 20 min at 4 °C after washing one time with PBS. The concentration of protein was determined with a BCA protein assay (Beyotime). The amount of protein was equally added for later gel electrophoresis and blotted onto nitrocellulose membranes (Beyotime). After being blocked by 10% nonfat milk for 2 h, the membranes were incubated with the primary antibodies including YAP, α-SMA and β-tubulin (Proteintech) overnight at 4 °C. Then, the membranes were washed three times (each time for 10 min) with Tris Buffered Saline with Tween (TBST) and further incubated with the secondary antibody (Proteintech) for 1.5 h at RT. After washing three times with TBST, the bands were imaged. All antibodies were diluted 2000 times before used.

### Neutralization capability of PF-MNs towards different inflammatory cytokines

Mouse IFN-γ (Biolegend) was incubated with HP MNs, PF-MNs, and PEG MNs at 37 °C at a concentration of 1000 pg/mL in PBS. 100 μL of the supernatant was taken out at intervals of 0, 1, 3, 6, 12, and 24 h, and stored at −20 °C until quantification with the ELISA kit (Biolegend). Moreover, the capability of MNs to scavenge MCP-1, TNF-α, IL-1β, and IL-6 was also investigated by multiplex immunoassay after 24 h.

### In vitro immune regulation of PF-MNs

10^4^ RAW 264.7 cells were cultured in a 24-well plate and incubated at 37 °C for 24 h. Then, LPS (100 ng/mL), IFN-γ (100 ng/mL), and IFN-γ (100 ng/mL) after being incubated with PF-MNs for an hour were added to induce M1 polarization. 24 h later, the solutions were centrifuged at 350×*g* for 5 min at 4 °C. The supernatant was discarded and resuspended in 1 mL of flow cytometry buffer (2% FBS in PBS). Then the cells were analyzed with the method of flow cytometry (Beckman CytoFlex S).

### In vitro antibacterial activity of PF-MNs

To evaluate the antibacterial ability of PF-MNs, 1 mL of *MRSA*, *SA*, *E. coli*, and *PA* solutions (10^6^ CFU/mL) were added into 48-well plates respectively, and further treated with PBS, laser irradiation, antibiotics (ampicillin, 2 μg/mL), PF-MNs, VP with laser irradiation (30 μg/mL), and PF-MNs with laser irradiation, respectively. The laser irradiation was applied for 10 min (25 mW/cm^2^). After different treatments, the plate was incubated at 37 °C for 2 h. 10 μL of diluted solution was plated on the LB agar and incubated at 37 °C overnight before calculating the CFU^49^.

### Biofilm formation and anti-biofilm activity of PF-MNs

To evaluate the ability of PF-MNs to prevent biofilm formation, 1 mL of *MRSA* solution (10^7^ CFU/mL) was added into a 24-well plate per well with circle microscope cover glass. After incubation at 37 °C for 6 h, the medium was replaced with different agents: (1) Control group (fresh LB), (2) Laser group (fresh LB with laser irradiation), (3) Anti group (ampicillin dissolved in fresh LB, 2 μg/mL), (4) VP + L group (VP solution dissolved in fresh LB, 30 μg/mL), (5) PF-MN group (PF-MNs in fresh LB), and (6) PF-MN + L group (PF-MNs in fresh LB with laser irradiation)^39^. The laser involved groups were exposed under 690 nm laser irradiation for 10 min (25 mW/cm^2^). After different treatments, the plate was incubated at 37 °C for another 36 h. The plate was washed three times with PBS, and stained with 500 μL crystal violet (1 g/L) for 10 min after incubating with anhydrous methanol for 10 min. The stained biofilms were washed three times with PBS, and photographed after air-dried. The absorbance at 570 nm was obtained using the Hybrid Multi-Mode Reader when the stained biofilms were dissolved in 500 μL of 95% ethanol. Meanwhile, biofilms with different treatments were stained with DMAO in live & dead bacterial staining kit for 20 min, and then imaged by CLSM (Olympus FV1000) to obtain 3D structures of biofilms using ImageJ. Besides, biofilms treated with the above method, were fixed with 4% paraformaldehyde (PFA, Solarbio) for 10 min, and then dehydrated with ethanol of 50%, 60%, 70%, 80%, 90%, and 100% in sequence over 10 min^50^. Finally, the samples were observed by SEM.

To evaluate the ability of destructing mature biofilms by PF-MNs, 1 mL of *MRSA* solution (10^7^ CFU/mL) was added into a 24-well plate per well with circle microscope cover glass and incubated at 37 °C for 48 h to obtain mature biofilms. Fresh LB was replaced every 24 h^51^. Then, the medium was replaced with the above agents. After different treatments, the plate was incubated at 37 °C for another 24 h. The biofilms were then assessed using crystal violet staining, live & dead staining and SEM in the same way above. The anti-biofilm activity of PF-MNs on *PA*-formed biofilms was also investigated with the above methods.

### Acute wound healing efficacy

Male BALB/c mice (6-8 weeks) were obtained from Shanghai Slac Laboratory Animal Co., Ltd. All animal procedures were approved by the Laboratory Animal Welfare and Ethics Committee of Zhejiang University (ZJU20220079). The mice were randomly divided into four groups (15 mice per group) and anesthetized with isoflurane (RWD). Then two round wounds with a diameter of 8 mm were made on the back of each mouse after depilation and then treated with 10 μL of PBS (Control group), 10 μL of 3 mg/mL VP (VP group), PF-MNs without VP (blank PF-MN group), and PF-MNs loaded with 30 μg VP (PF-MN group), respectively (day 0). The mice were individually housed in cages to prevent the interference among mice. A medical tape was applied on the back of MN patches to further immobilize them on the mice. And the mice of other groups were also stuck with the medical tape as control. The MNs were removed on day 2. On days 3, 7, and 14, the wounds were photographed and wound areas were measured. Meanwhile, the wounds after different treatments were collected and stored in 4% PFA for H&E staining. All the histological slices were recorded using a virtual slide microscope (Olympus VS200) and the epidermal thickness of wounds was analyzed using ImageJ. Besides, the wounds after different treatments on day 3 were collected in flow cytometry buffer for flow cytometry analysis.

### Diabetic mouse infection model

The diabetic mouse model was established by intraperitoneal injection of 50 mg/kg STZ (10 mg/mL) to male BALB/c mice (6–8 weeks) for five consecutive days^52^. STZ was dissolved in citric acid sodium citrate buffer (pH 4.4), which was placed on ice till injection. The injection was finished within 30 min. A week later, the mice were determined as diabetic ones if the blood glucose >16.7 mmol/L. The diabetic mice were anesthetized with isoflurane, and two round wounds with the diameter of 8 mm were made on the back of each mouse after depilation. Then, the wounds were infected with 10^7^ CFU of *MRSA* (10^9^ CFU/mL). Two days later, the diabetic mouse infection model was established.

### In vivo anti-biofilm activity of PF-MNs

The diabetic mice infected with *MRSA* were randomly divided into six groups (12 mice per group). The mice were treated with 10 μL of PBS (Control group), 10 μL of ampicillin (200 μg/mL) (Anti group), 10 μL of VP (3 mg/mL) with laser irradiation (VP + L group), non-degradable MNs with laser irradiation (NMN + L group), PF-MNs (PF-MN group), and PF-MNs with laser irradiation (PF-MN + L group) (day 0). The laser irradiation was applied for 10 min (690 nm, 25 mW/cm^2^). All the MNs were loaded with VP (30 μg) and removed on day 2. The mice were individually housed in cages and the MNs were immobilized with a medical tape as the method above. On days 3, 7, and 14, the wounds were photographed and the wound areas were measured. Meanwhile, the wounds after different treatments were collected and stored in 4% PFA for H&E staining, Masson Trichrome staining, Sirius Red staining and immunofluorescence staining (including F4/80, CD86, CD206, CD31, and α-SMA)^40^. All the histological slices were recorded using a virtual slide microscope (Olympus VS200) and statistical analysis was analyzed using ImageJ. On day 3, the wounds were collected in PBS (500 μL) and then homogenized with a homogenizer (KZ-III-FP). The homogenates were divided into two parts: one part was for bacterial plate counting (10 μL), and the other one was for inflammatory factors quantitative analysis. Serially diluted homogenates were plated on LB agar to quantify the bacterial colonies. Then the homogenates were centrifuged at 10,000 rpm for 10 min at 4 °C. The supernatants were taken out to detect the inflammatory factors by multiplex immunoassay (Biolegend), including IFN-γ, TNF-α, MCP-1, IL-1β, and IL-6.

### Flow cytometry analysis of wounds from mice

For healthy mice and diabetic mice, the wound tissues were collected on day 3 and chopped. Then, the tissues were digested with hyaluronidase/collagenase II (5 mL) at 37 °C for 30 min and terminated by flow cytometry buffer. The solutions were centrifuged at 350×*g* for 5 min at 4 °C before being plunged through a 40 μm filter to obtain a single cell suspension. Polyclonal antibodies for staining including: Anti-CD16/32, Anti-F4/80, Anti-CD86, Anti-CD206, Anti-CD45, Anti-CD4, and Anti-FoxP3 (Biolegend). All the amounts of antibodies for staining were followed the manufacturer’s instructions. All data were acquired on a CytoFLEX flow cytometer and analyzed with FlowJo10.

### In vivo biosafety assessment

To estimate the biosafety of PF-MNs, the blood of diabetic mice with *MRSA* infection was collected for further analysis after different treatments on day 3. Besides, the major organs on day 14 (heart, liver, spleen, lung, and kidney) of mice were also collected for H&E staining and all the histological slices were recorded using a virtual slide microscope (Olympus VS200).

### Transcriptome sequencing and data analysis

The wound tissues of diabetic mice on day 7 were collected and washed with saline. Then, the tissues were stored at –80 °C before sequencing (LC-Bio Technologies in Hangzhou Co., Ltd.). Differential expression analysis was conducted using R Bioconductor package “edgeR”. The R package “ClusterProfiler” (version 4.0.5) was applied for GO enrichment and KEGG pathway enrichment analysis.

### Cytotoxicity assay

Cytotoxicity of different concentrations of VP was evaluated using the CCK-8 assay. Briefly, 10^4^ L929 cells were seeded in a 24-well plate at 37 °C for 24 h. And then they were exposed to different concentrations of VP and PF-MNs for 24 h, in which cells with PF-MNs were exposed to 690 nm laser irradiation (25 mW/cm^2^, 10 min). The cells were then washed with PBS and incubated with complete media containing 10% CCK-8 for 2 h at 37 °C, before reading the absorbance at 450 nm using the Hybrid Multi-Mode Reader.

### Mitigation of scar formation by PF-MNs

New Zealand white male rabbits (3–4 months) were anaesthetized by intravenous injection of pentobarbital (30 mg/kg). Then each ear received three wounds using an 8 mm biopsy punch and the perichondrium was completely removed using a scalpel. Then, the wounds were treated with PBS (10 μL), VP (10 μL, 3 mg/mL), blank PF-MNs, and PF-MNs, respectively. The MNs were stabilized by a sterilized bandage and removed after two days. The wounds were photographed during the treatments and the scars were collected on day 30 for H&E staining, Masson Trichrome staining and Sirius Red staining. All the histological slices were recorded using a virtual slide microscope (Olympus VS200) and the SEI, percentage of collagen, and type I/III collagen ratio were analyzed by ImageJ.

### Photodynamic therapy with PF-MNs for the treatment of HS

The rabbit ear HS model was established according to the previously method^43^. In detail, after anaesthetization with pentobarbital (30 mg/kg), three wounds were established using an 8 mm biopsy punch on each ear. The perichondrium was completely removed using a scalpel. 30 days later, the wounds were completely healed and hypertrophic scars were formed. Then the rabbits were randomly divided into four groups: Control group (no treatment), Laser group (laser irradiation), PF-MN group (PF-MNs), and PF-MN + L group (PF-MNs with laser irradiation). The laser irradiation was applied for 10 min (25 mW/cm^2^) and the MNs were immobilized by a sterilized bandage for two days. The scars were photographed during the treatments and the wounds were collected on day 15, 25, and 35 for H&E staining, Masson Trichrome staining, and Sirius Red staining. All the histological slices were recorded using a virtual slide microscope (Olympus VS200) and the SEI, percentage of collagen, and type I/III collagen ratio were analyzed by ImageJ.

### Statistical analysis

The GraphPad Prism software (version 8.2.1) and ImageJ software (version 2.1.0) were used for the statistical analysis. All data were obtained from at least three independent experiments with at least three parallel samples per condition in each experiment and are expressed as mean ± standard deviations (SD). Multiple comparisons were performed using one-way two-sided analysis of variance (ANOVA) with Tukey’s multiple comparison test. A probability value of *P* < 0.05 was considered statistically significant (*P < 0.05, **P < 0.01, ***P < 0.001, ****P < 0.0001).

### Reporting summary

Further information on research design is available in the Nature Portfolio Reporting Summary linked to this article.

## Supplementary information

Supplementary information

Reporting Summary

## Data Availability

The authors declare that all the data supporting the findings of this study are available within the article and supplementary information and from the corresponding authors upon request. Source data are provided as a Source Data file.
