# Supplementary material for: Scarless wound healing programmed by core-shell microneedles
Source: Nat Commun. 2023 Jun 10;14:3431. doi: 10.1038/s41467-023-39129-6 (PMC10257705; doi:10.1038/s41467-023-39129-6)
Supplement: Supplementary file 1 — Supplementary information [file 41467_2023_39129_MOESM1_ESM.pdf]

Supplementary Information

Scarless Wound Healing Programmed by Core-Shell Microneedles

Ying Zhang[1,+], Shenqiang Wang[1,+]*, Yinxian Yang[1], Sheng Zhao[1], Jiahuan You[1], Junxia Wang[1], Jingwei Cai[2], Hao Wang[1], Jie Wang[3], Wei Zhang[3], Jicheng Yu[1,2,4,5,6], Chunmao Han[3], Yuqi Zhang[1,3,6]*, Zhen Gu[1,2,4,5,6,7]*

[1]Key Laboratory for Advanced Drug Delivery Systems of Zhejiang Province, College of Pharmaceutical Sciences, Zhejiang University
Hangzhou 310058, China
[2]Department of General Surgery, Sir Run Run Shaw Hospital, School of Medicine, Zhejiang University
Hangzhou, 310016, China
[3]Department of Burns and Wound Care Center, the Second Affiliated Hospital, College of Medicine, Zhejiang University
Hangzhou 310009, China
[4]Jinhua Institute of Zhejiang University
Jinhua 321299, China
[5]Liangzhu Laboratory, Zhejiang University Medical Center
Hangzhou 311121, China
[6]National Key Laboratory of Advanced Drug Delivery and Release Systems, Zhejiang University
Hangzhou 310058, China
[7]MOE Key Laboratory of Macromolecular Synthesis and Functionalization, Department of Polymer Science and Engineering, Zhejiang University
Hangzhou 310027, China

[+]These authors contributed equally: Ying Zhang, Shenqiang Wang

*E-mail: guzhen@zju.edu.cn (Z.G.); yqzhang21@zju.edu.cn (Y.Z.); wangshenqiang@zju.edu.cn (S.W.)

This PDF file includes:

Supplementary Fig. 1 Synthesis route of TSPBA and ^{1}H-NMR of TSPBA before and after oxidization in 10 mM H_2O_2 for 1 h.

Supplementary Fig. 2 Synthesis route of m-heparin and ^{1}H-NMR of heparin and m-heparin.

Supplementary Fig. 3 A 3D Confocal image of Rhodamine B-labelled PF-MN shell.

Supplementary Fig. 4 Absorption spectra of VP before and after irradiation with 690 nm laser for 10 min.

Supplementary Fig. 5 The release profiles of VP from MN shell with different ratios of PVA:TSPBA (1:1, 2:1, 3:1, and 4:1) in 0.1 mM H_2O_2.

Supplementary Fig. 6 Binding amounts of different inflammatory factors.

Supplementary Fig. 7 Weight remaining ratio of cHP incubated in PBS.

Supplementary Fig. 8 Evaluation of antibacterial and anti-biofilm activities by PF-MNs.

Supplementary Fig. 9 Accelerated wound healing with PF-MNs in acute wounds.

Supplementary Fig. 10 Gating strategies for macrophages and Treg cells analysis by flow cytometry in acute wounds.

Supplementary Fig. 11 Gating strategies for macrophages and Treg cells analysis by flow cytometry in wounds of diabetic mice.

Supplementary Fig. 12 Blood routine on day 3 of diabetic mice received different treatments.

Supplementary Fig. 13 Immunofluorescence staining of wounds on day 7.

Supplementary Fig. 14 Histological analysis of wounds from diabetic mice on different days.

Supplementary Fig. 15 Blood biochemistry analysis on day 3 of diabetic mice received different treatments.

Supplementary Fig. 16 Biocompatibility of PF-MNs in vivo.

Supplementary Fig. 17 Mechanistic analysis of wound healing with PF-MN treatment.

Supplementary Fig. 18 Apoptosis analysis of fibroblasts.

Supplementary Fig. 19 Histological analysis of HS after PF-MN treatment on day 25 and day 35.

Supplementary Figures

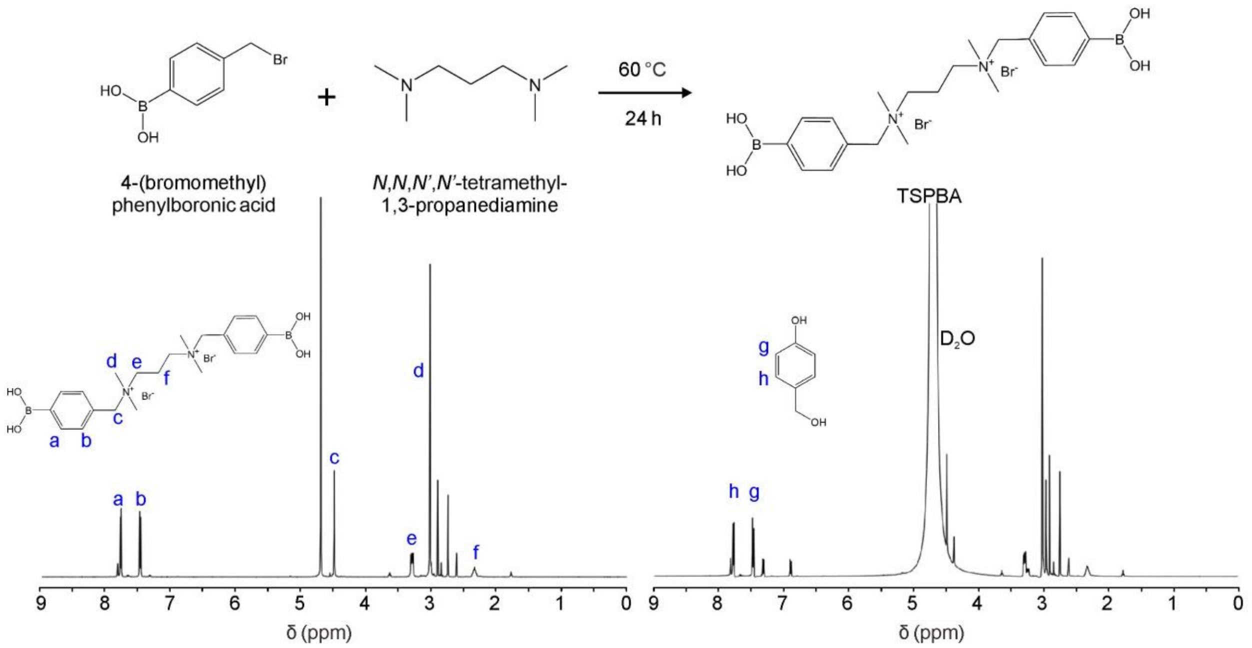

Supplementary Fig. 1 | Synthesis route of TSPBA and ^{1}H-NMR of TSPBA before and after oxidization in 10 mM H_2O_2 for 1 h. ^{1}H NMR (500 MHz, D_2O) of TSPBA: δ 7.77 (d, 4H), 7.47 (d, 4H), 4.49 (s, 4H), 3.30 (m, 4H), 3.02 (s, 12H), 2.34 (m, 2H).

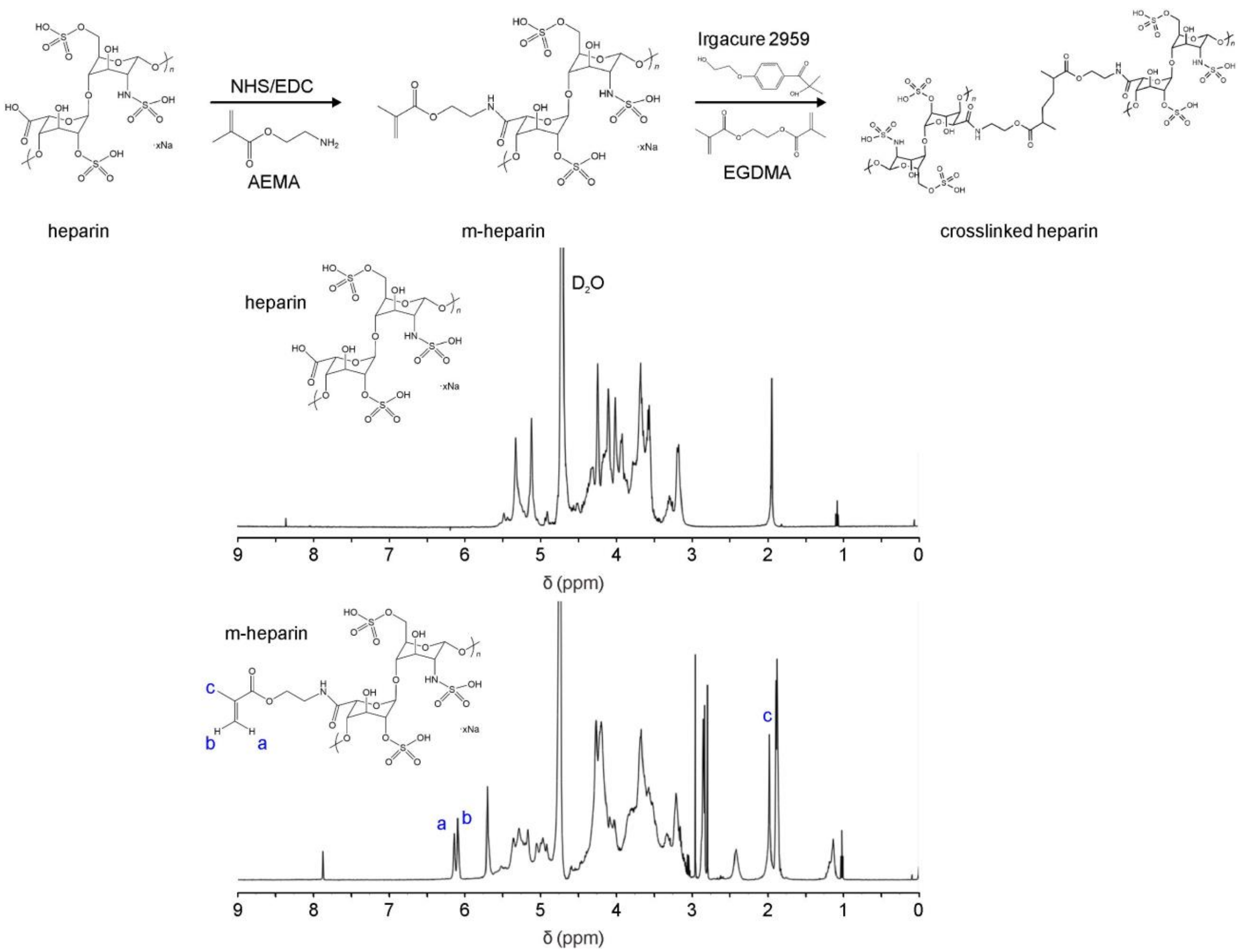

Supplementary Fig. 2 | Synthesis route of m-heparin and ^{1}H-NMR of heparin and m-heparin. ^{1}H NMR (500 MHz, D_2O) of m-heparin: δ 6.13 (s, 1H), 6.09 (s, 1H), 1.98 (d, 3H).

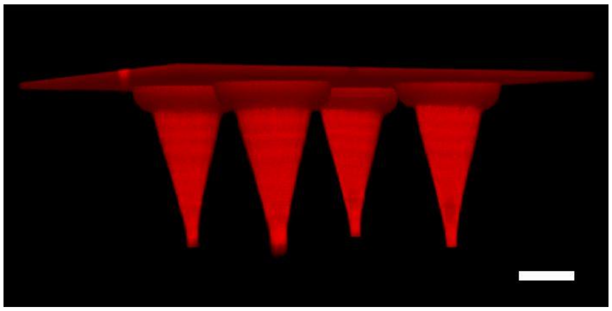

Supplementary Fig. 3 | A 3D Confocal image of Rhodamine B-labelled PF-MN shell. Red: Rhodamine B. Scale bar, 200 µm.

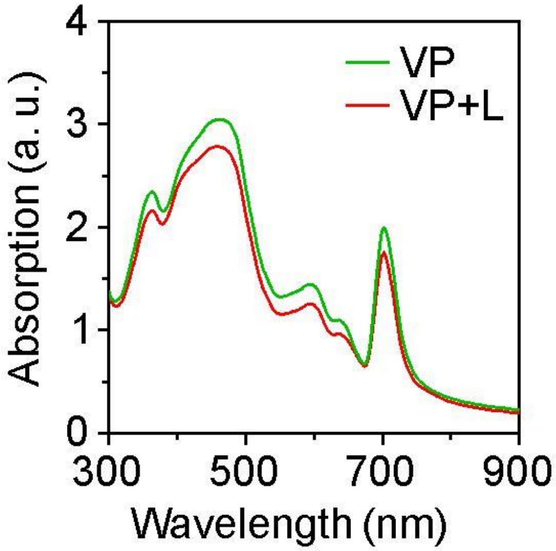

Supplementary Fig. 4 | Absorption spectra of VP before and after irradiation with 690 nm laser for 10 min.

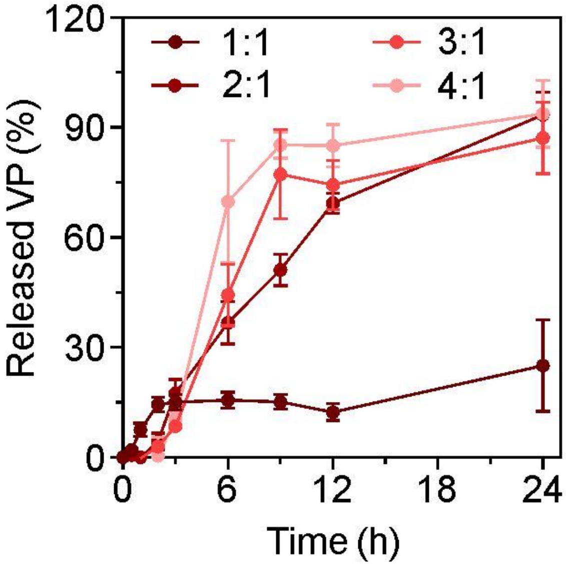

Supplementary Fig. 5 | The release profiles of VP from MN shells with different ratios of PVA:TSPBA (1:1, 2:1, 3:1, and 4:1) in 0.1 mM H_2O_2 (n = 3 independent samples). Data are presented as mean ± SD.

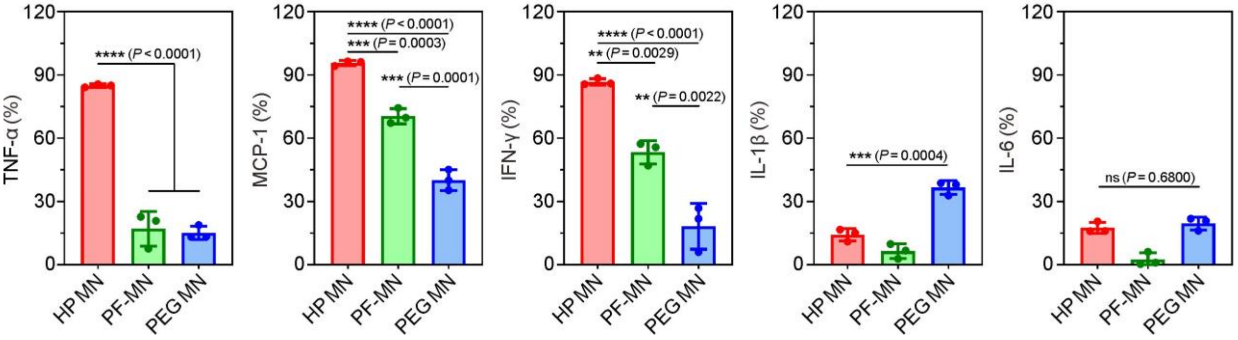

Supplementary Fig. 6 | Scavenged pro-inflammatory cytokines after incubation with heparin (HP) MNs, PF-MNs, and PEG MNs for 24 h, including TNF-α, MCP-1, IFN-γ, IL-1β, and IL-6 (n = 3 independent samples). Data are presented as mean ± SD and statistical significance was analyzed via one-way ANOVA with Tukey's multiple comparison test. P value: ** $P < 0.01$, *** $P < 0.001$, **** $P < 0.0001$.

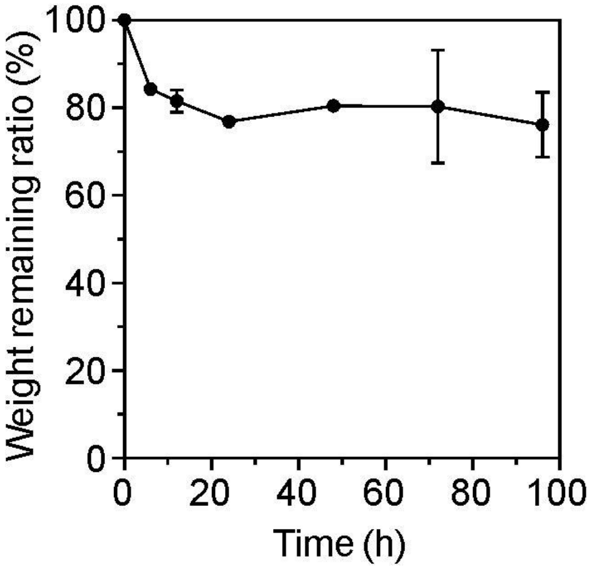

Supplementary Fig. 7 | Weight remaining ratio of cHP incubated in PBS (n = 3 independent samples). Data are presented as mean ± SD.

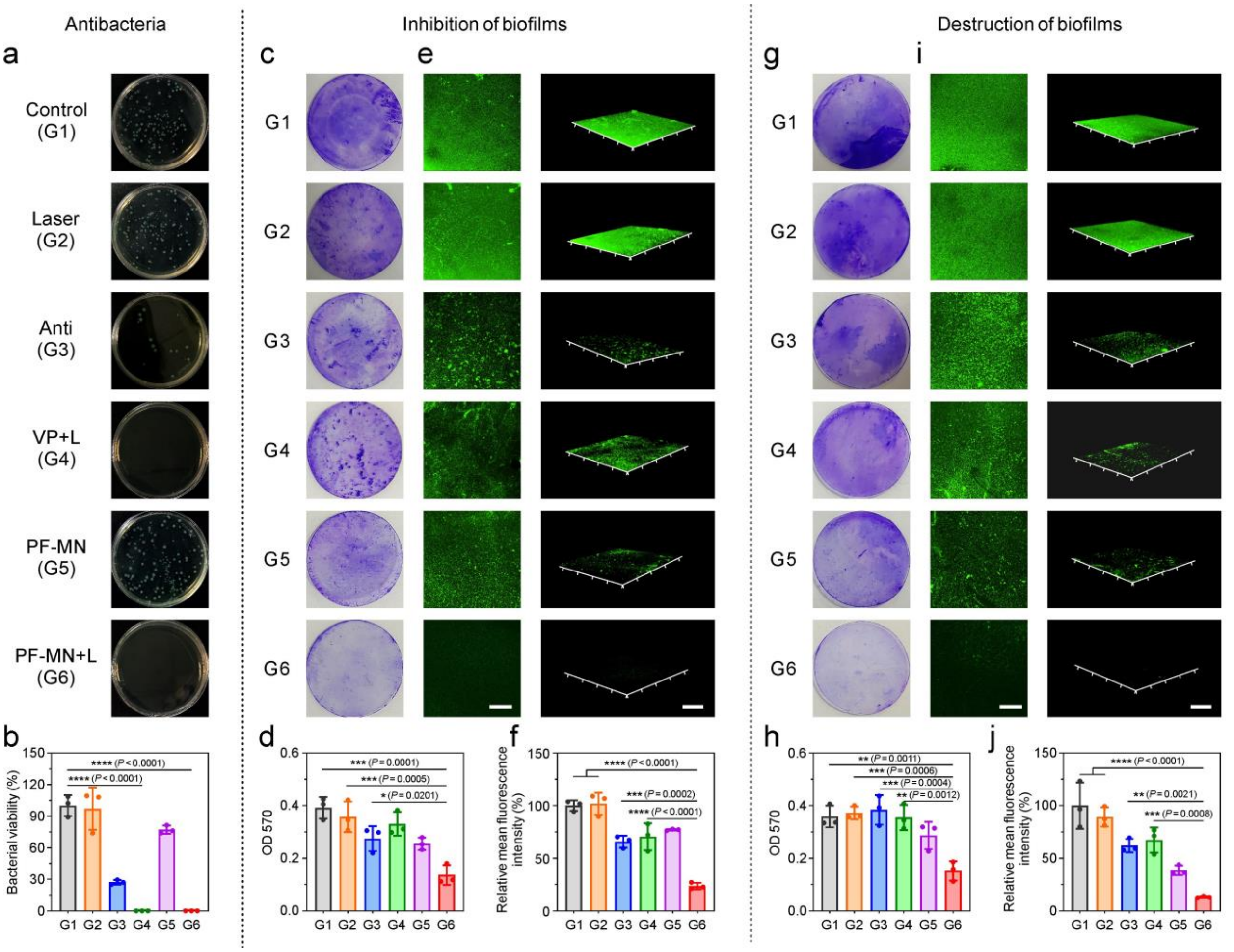

Supplementary Fig. 8 | Evaluation of antibacterial and anti-biofilm activities by PF-MNs. a Photographs of bacterial colonies of *PA* on LB agar plates after different treatments. **b** Corresponding bacterial viability of PA after different treatments (n = 3 independent samples). **c** Photographs of the immature biofilms stained by crystal violet, showing the inhibitory effects of PF-MNs. **d** Corresponding OD 570 values of immature biofilms of each group (n = 3 independent samples). **e** Confocal images and corresponding 3D images of DMAO-stained (green) immature biofilms after different treatments. Scale bar, 200 μm. **f** Corresponding fluorescence intensities of DMAO-stained (green) immature biofilms (n = 3 independent samples). **g** Photographs of the mature biofilms stained by crystal violet, showing the destructive effects of PF-MNs. **h** Corresponding OD 570 values of mature biofilms (n = 3 independent samples). **i** Confocal images

and corresponding 3D images stained with DMAO of mature biofilms in different groups. Scale bar, 200 μm. **j** Corresponding fluorescence intensities of DMAO-stained mature biofilms (n = 3 independent samples). L: Laser. Three independent experiments were performed and representative results are shown in **c**, **e**, **g**, and **i**. Data are presented as mean ± SD and statistical significance was analyzed via one-way ANOVA with Tukey's multiple comparison test. P value: * $P < 0.5$, ** $P < 0.01$, *** $P < 0.001$, **** $P < 0.0001$.

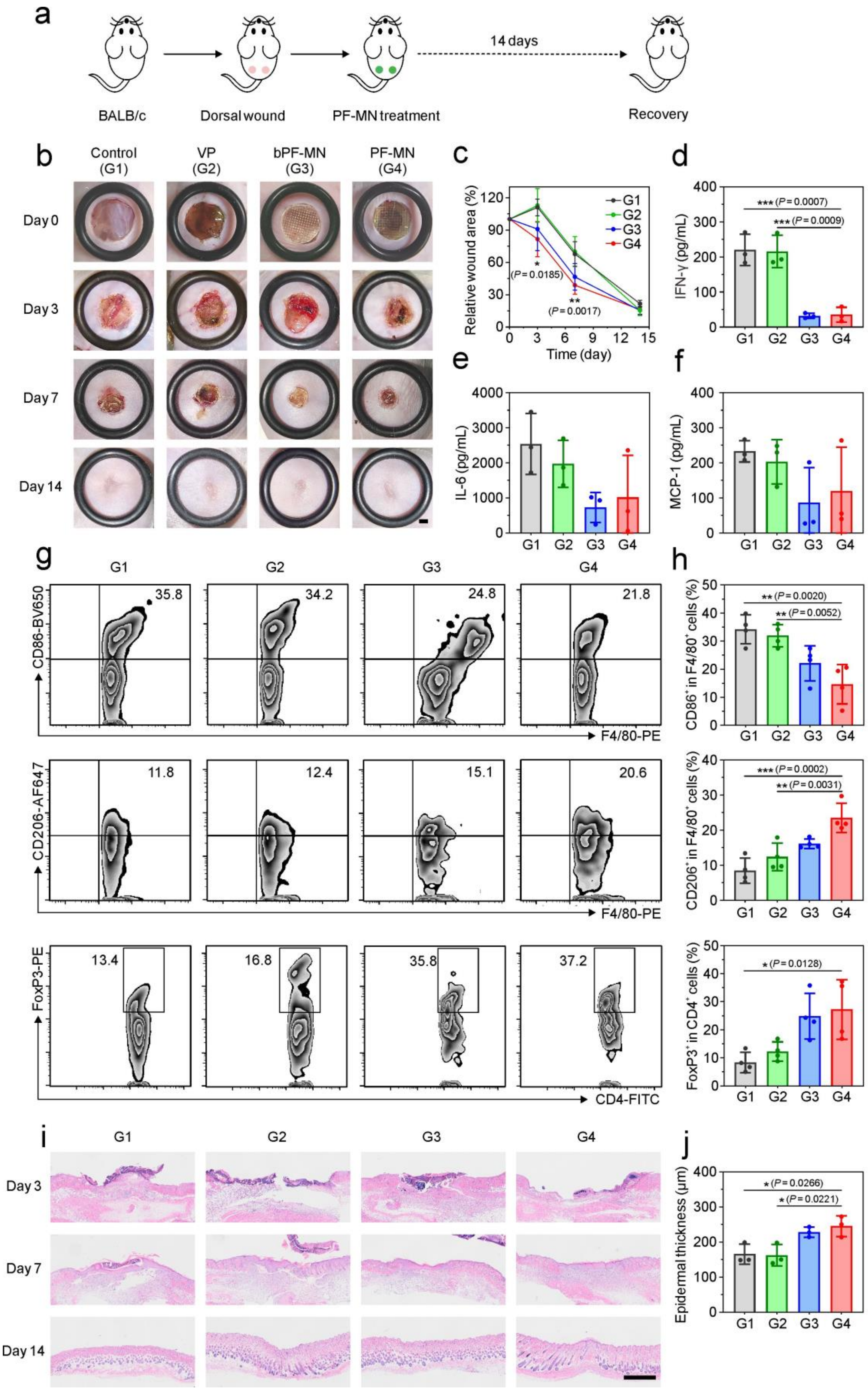
a
14 days
BALB/c
Dorsal wound
PF-MN treatment
Recovery
b
Control (G1)
VP (G2)
bPF-MN (G3)
PF-MN (G4)
Day 0
Day 3
Day 7
Day 14
c
Relative wound area (%)
Time (day)
G1
G2
G3
G4
(P = 0.0185)
(P = 0.0017)
d
IFN-γ (pg/mL)
*** (P = 0.0007)
*** (P = 0.0009)
e
IL-6 (pg/mL)
f
MCP-1 (pg/mL)
g
CD86-BV650
F4/80-PE
35.8
34.2
24.8
21.8
CD206-AF647
11.8
12.4
15.1
20.6
FoxP3-PE
CD4-FITC
13.4
16.8
35.8
37.2
h
CD86+ in F4/80+ cells (%)
** (P = 0.0020)
** (P = 0.0052)
CD206+ in F4/80+ cells (%)
*** (P = 0.0002)
** (P = 0.0031)
FoxP3+ in CD4+ cells (%)
* (P = 0.0128)
i
j
Epidermal thickness (μm)
* (P = 0.0266)
* (P = 0.0221)

Supplementary Fig. 9 | Accelerated wound healing with PF-MNs in acute wounds. a Experimental schematic indicating PF-MN treatment process on BALB/c mice. **b** Photographs of wounds of BALB/c mice after treatment with PBS, VP, blank PF-MNs (bPF-MNs), and PF-MNs. Scale bar, 2 mm. **c** Quantitative analysis of the relative wound area after different treatments (n = 6 biologically independent samples). **d-f** Quantitative analysis of the levels of IFN-γ (**d**), IL-6 (**e**), and MCP-1 (**f**) from the wounds on day 3 (n = 3 biologically independent samples). **g-h** Representative flow cytometry plots (**g**) of macrophages and regulatory T cells and corresponding quantitative analysis (**h**) from wounds on day 3 (n = 4 biologically independent samples). **i** H&E staining images of wounds on day 3, 7, and 14. Scale bar, 1 mm. **j** Statistical analysis of epidermal thickness on day 7 (n = 3 biologically independent samples). Three independent experiments were performed and representative results are shown in **i**. Data are presented as mean ± SD and statistical significance was analyzed via one-way ANOVA with Tukey's multiple comparison test. P value: * $P < 0.05$, ** $P < 0.01$, *** $P < 0.001$.

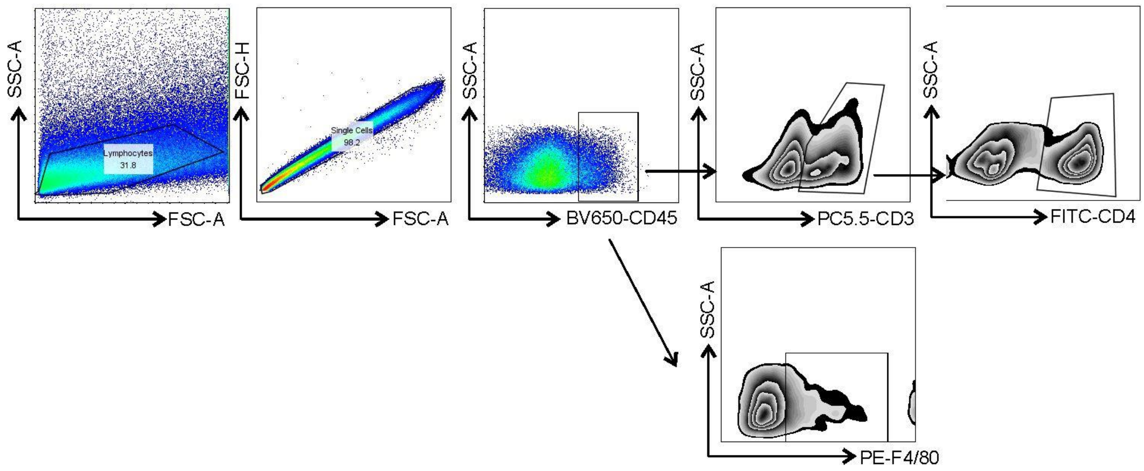

Supplementary Fig. 10 | Gating strategies for macrophage and Treg cell analysis by flow cytometry in acute wounds.

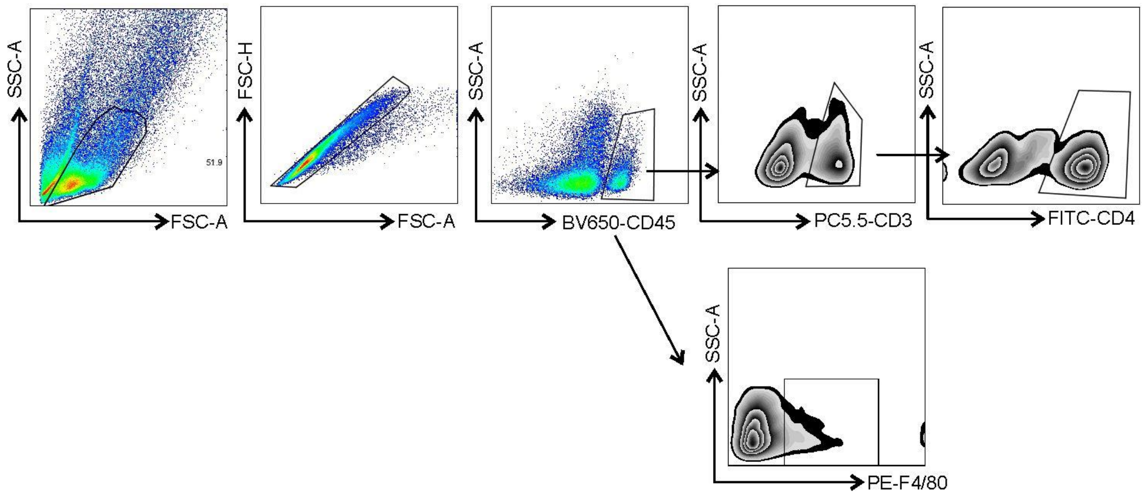

Supplementary Fig. 11 | Gating strategies for macrophage and Treg cell analysis by flow cytometry in diabetic wounds.

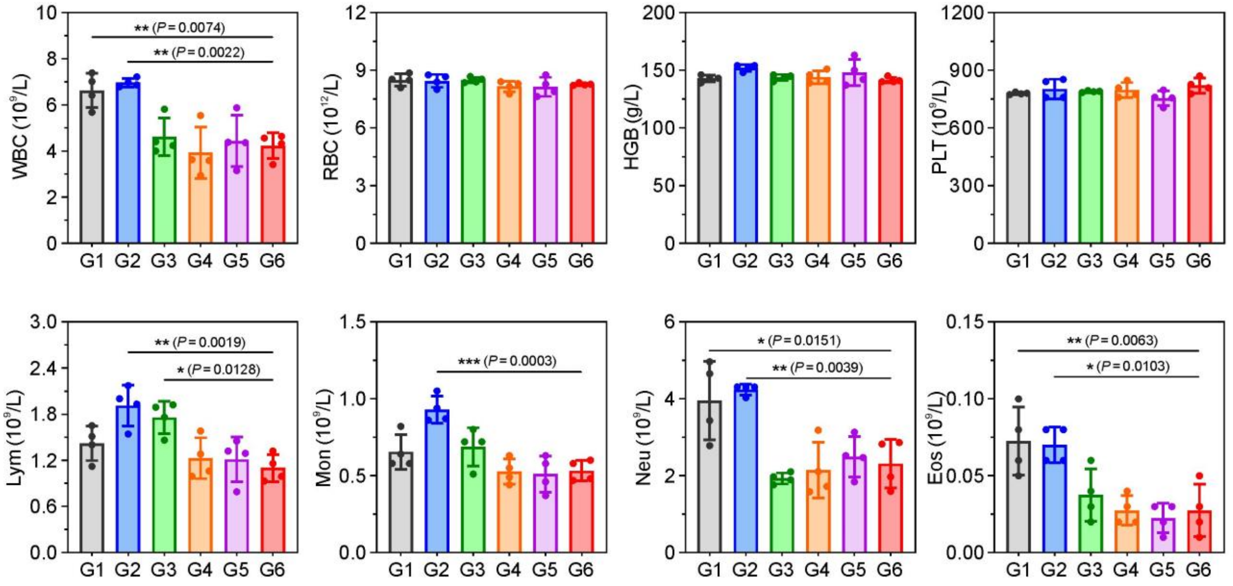

Supplementary Fig. 12 | Blood routine on day 3 of diabetic mice received different treatments (G1: Control, G2: Anti, G3: VP+L, G4: NMN+L, G5: PF-MN, G6: PF-MN+L) (*n* = 4 biologically independent animals). WBC: white blood cell, RBC: red blood cell, HGB: hemoglobin, PLT: platelet, Lym: lymphocyte, Mon: monocyte, Neu: neutrophil, Eos: eosinophil. Data are presented as mean ± SD and statistical significance was analyzed via one-way ANOVA with Tukey's multiple comparison test. *P* value: * $P < 0.05$, ** $P < 0.01$, *** $P < 0.001$.

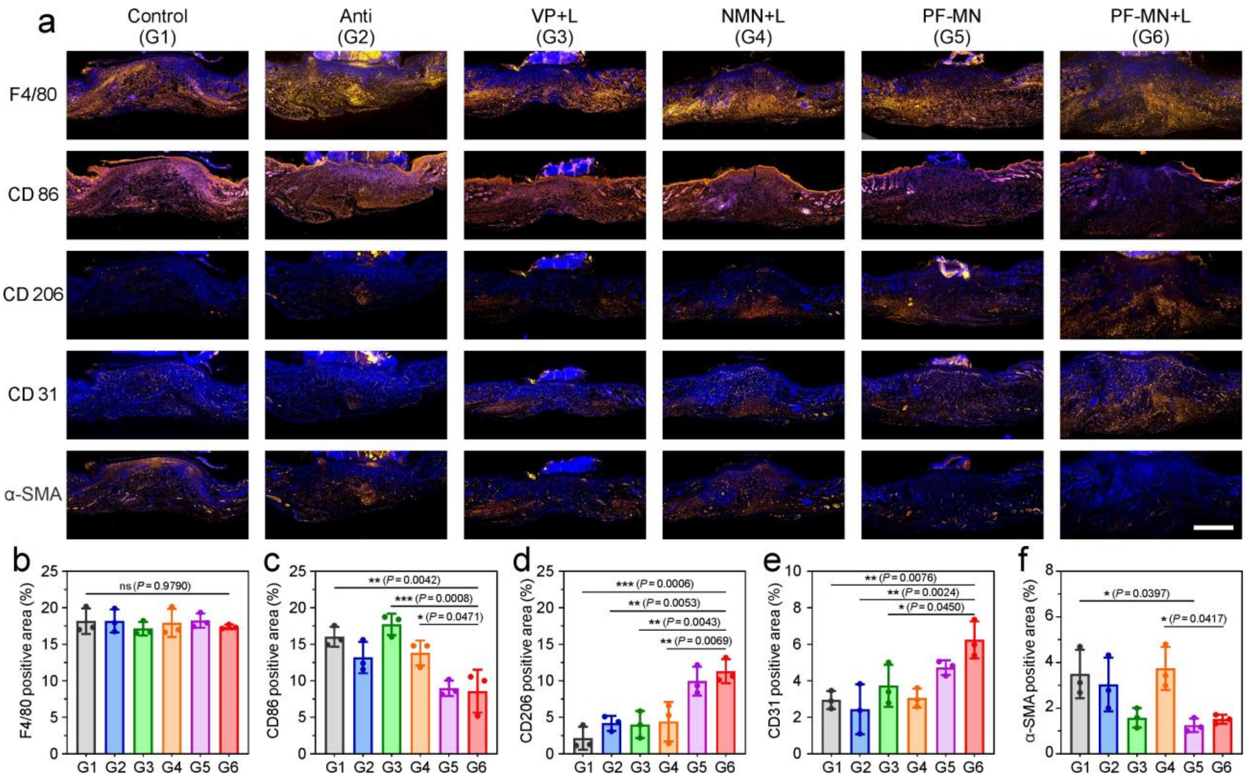

Supplementary Fig. 13 | Immunofluorescence staining of wounds on day 7. a Representative fluorescence images from wounds in diabetic mice. Scale bar, 1 mm. **b-f** Statistical analysis of F4/80 (**b**), CD86 (**c**), CD206 (**d**), CD31 (**e**), and α-SMA (red) (**f**) relative fluorescence areas (n = 3 biologically independent samples). Three independent experiments were performed and representative results are shown in **a**. Data are presented as mean ± SD and statistical significance was analyzed via one-way ANOVA with Tukey's multiple comparison test. P value: * $P < 0.05$, ** $P < 0.01$, *** $P < 0.001$.

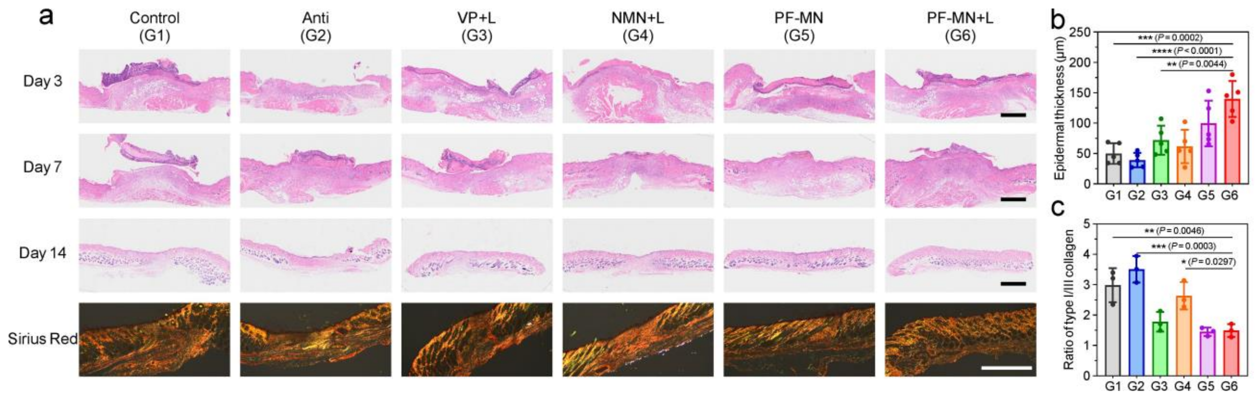

Supplementary Fig. 14 | Histological analysis of wounds from diabetic mice on different days. **a** Representative H&E staining images of wounds on day 3, 7, and 14 and Sirius Red staining images on day 14. Scale bar, 1 mm. **b** Corresponding statistical analysis of epidermal thickness on day 7 (n = 5 biologically independent samples). **c** The ratio of type I/III collagen on day 14 analyzed from the Sirius Red staining images (n = 3 biologically independent samples). Three independent experiments were performed and representative results are shown in **a**. Data are presented as mean ± SD and statistical significance was analyzed via one-way ANOVA with Tukey's multiple comparison test. P value: * $P < 0.05$, ** $P < 0.01$, *** $P < 0.001$, **** $P < 0.0001$.

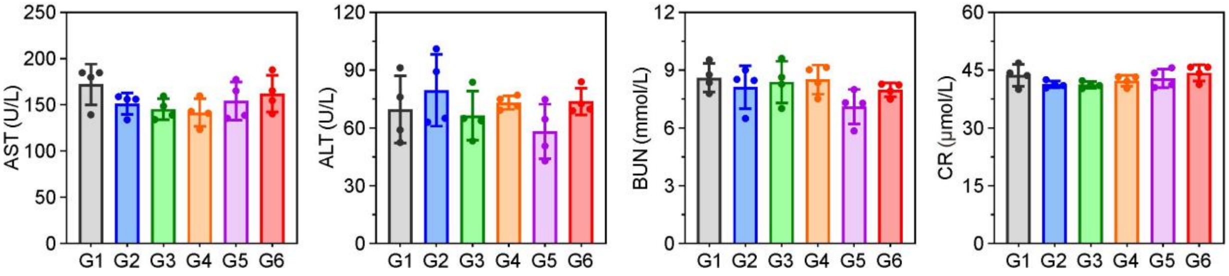

Supplementary Fig. 15 | Blood biochemistry analysis on day 3 of diabetic mice received different treatments (G1: Control, G2: Anti, G3: VP+L, G4: NMN+L, G5: PF-MN, G6: PF-MN+L) (*n* = 4 biologically independent animals). AST: aspartate transferase, ALT: alanine transferase, BUN: blood urea nitrogen, CR: creatinine. Data are presented as mean ± SD.

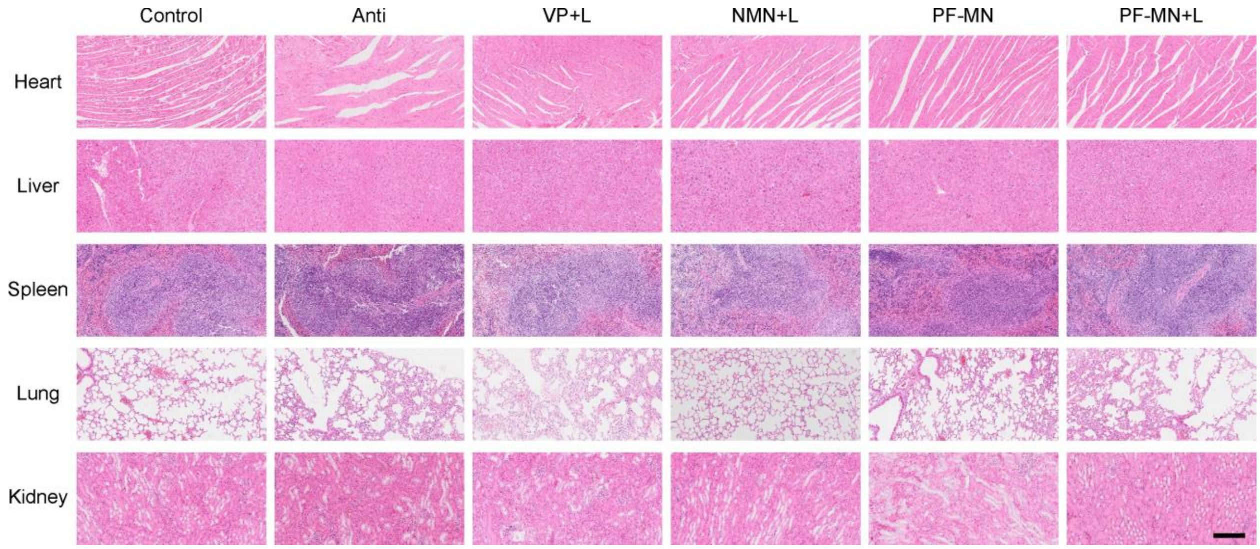

Supplementary Fig. 16 | Biocompatibility of PF-MNs in vivo. H&E staining images of major organs (heart, liver, spleen, lung and kidney) from diabetic mice on day 14. Scale bar, 200 μm. Three independent experiments were performed and representative results are shown.

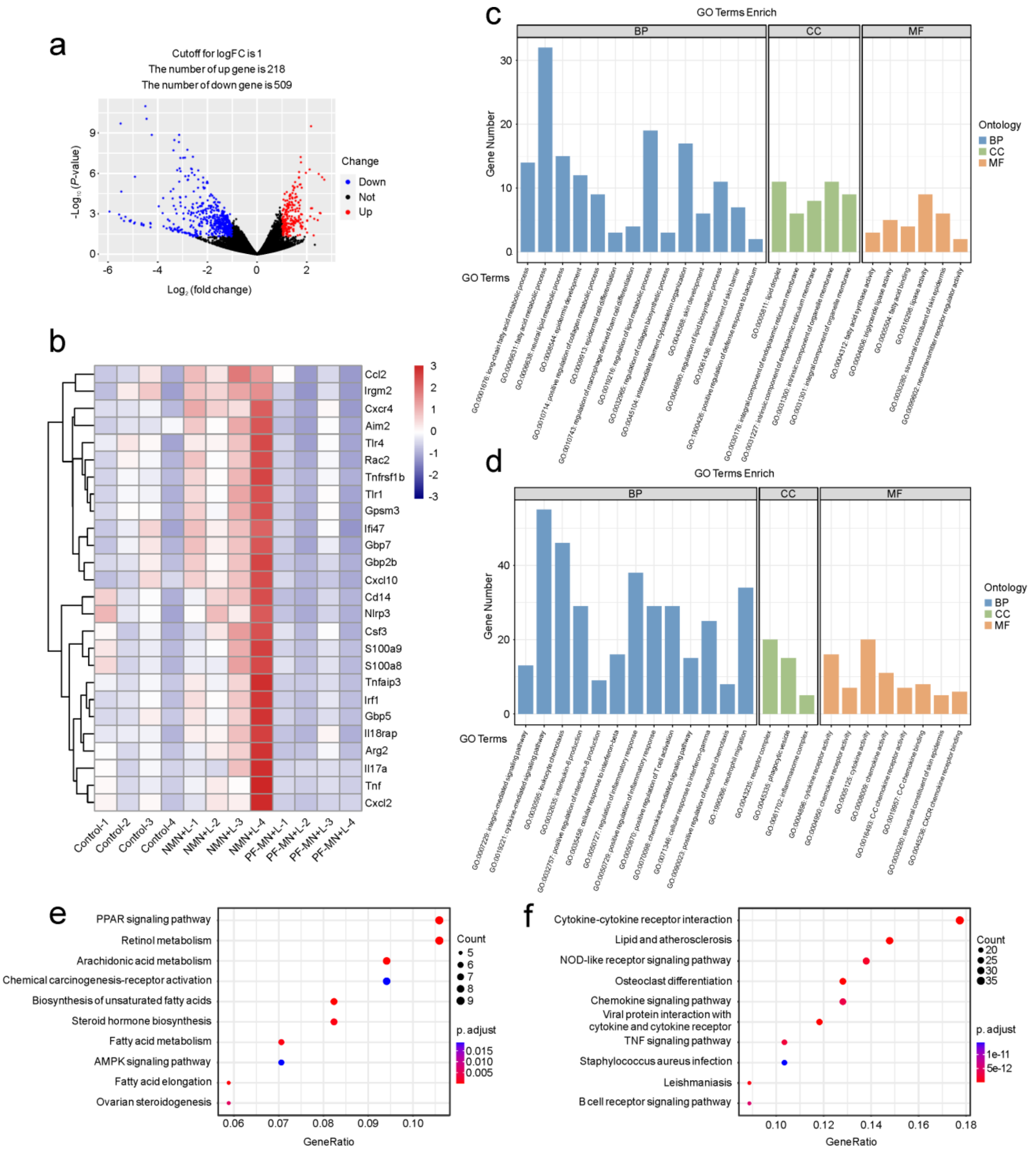

Supplementary Fig. 17 | Mechanistic analysis of wound healing with PF-MN treatment. a Volcano plot of transcriptomic analysis of differentially expressed genes (n = 4 biologically independent samples). **b** Heatmap analysis of differentially expressed genes. **c-d** GO analysis of differentially expressed genes. **e-f** Enriched KEGG pathways of PF-MN+L versus Control and

NMN+L. The size of the dots indicates the number of genes associated with indicated KEGG terms and the colour of the dots indicates the adjusted *P* values (p. adjust) calculated by one-sided Fisher's exact test with Benjamini-Hochberg correction.

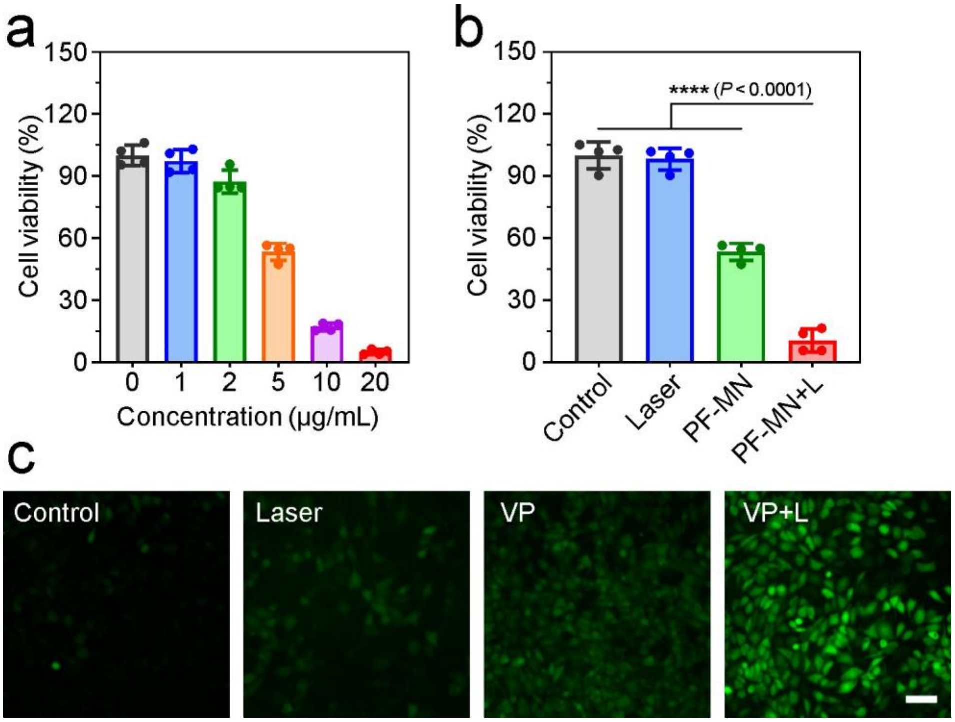

Supplementary Fig. 18 | Apoptosis analysis of fibroblasts. **a** Cytotoxicity of different concentrations of VP on fibroblasts (*n* = 4 independent samples). **b** Cell viability of fibroblasts after different treatments: laser irradiation, PF-MNs, or PF-MNs with laser irradiation (*n* = 4 independent samples). **c** Fluorescence images of fibroblasts treated with laser irradiation only, VP solution only (5 μg/mL), or VP solution (5 μg/mL) with laser irradiation, indicating the ROS generated by VP using DCFH-DA. Scale bar, 50 μm. Three independent experiments were performed and representative results are shown in **c**. Data are presented as mean ± SD and statistical significance was analyzed via one-way ANOVA with Tukey's multiple comparison test. *P* value: **** $P < 0.0001$.

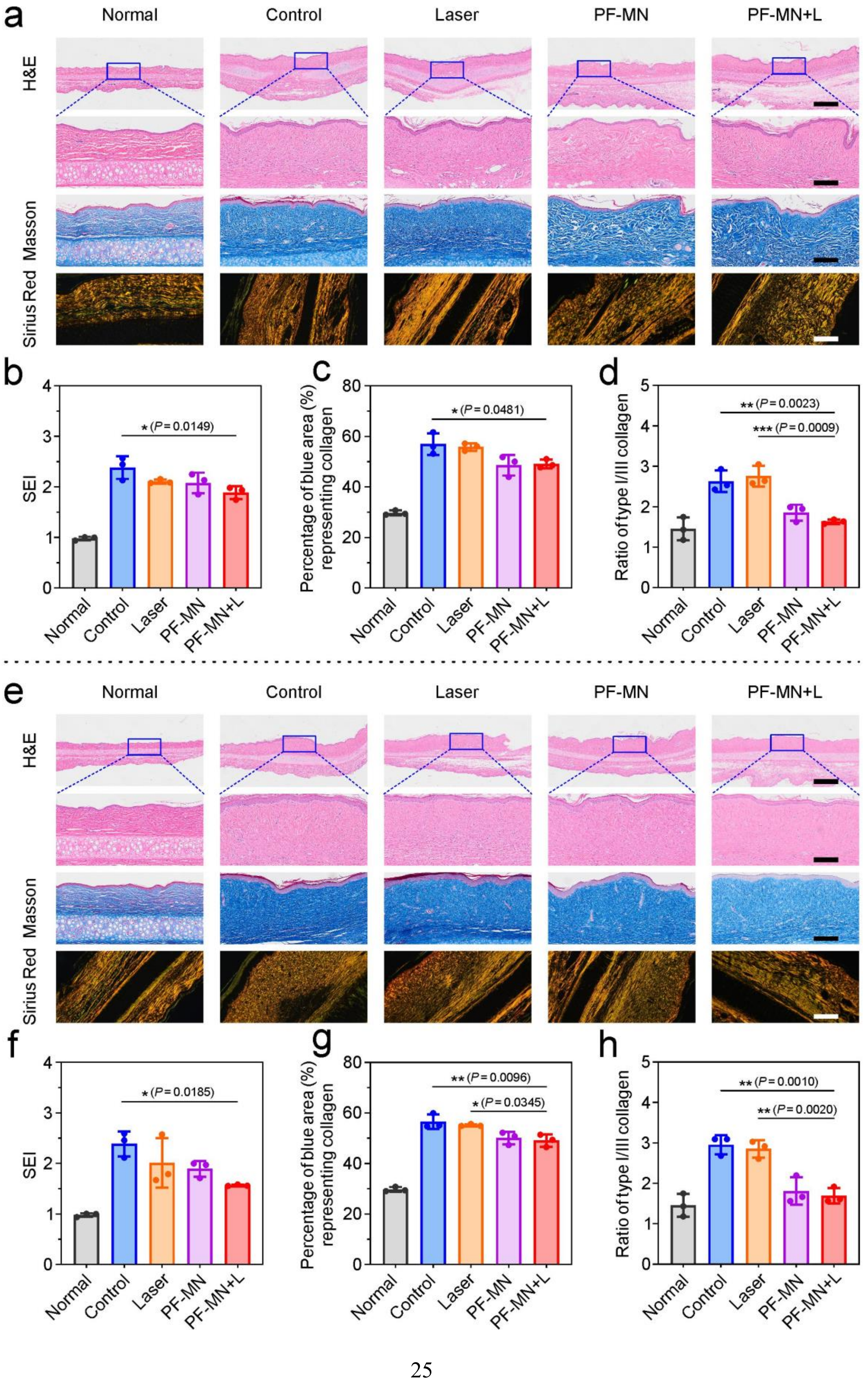
a
Normal
Control
Laser
PF-MN
PF-MN+L
H&E
Masson
Sirius Red
b
SEI
*(P = 0.0149)
c
Percentage of blue area (%) representing collagen
*(P = 0.0481)
d
Ratio of type I/III collagen
**(P = 0.0023)
***(P = 0.0009)
e
f
*(P = 0.0185)
g
**(P = 0.0096)
*(P = 0.0345)
h
**(P = 0.0010)
**(P = 0.0020)

1

Supplementary Fig. 19 | Histological analysis of HS after PF-MN treatment on day 25 and day 35. a Representative H&E staining, Masson Trichrome staining and Sirius Red staining images of HS on day 25. Scale bar, 1 mm in H&E staining image (top), 200 μm in H&E staining image (enlarged), Masson Trichrome staining and Sirius Red staining images. **b-d** Corresponding quantitative analysis of SEI (**b**), percentage of collagen (**c**), and type I/III collagen (**d**) of HS on day 25 (n = 3 biologically independent samples). **e** Representative H&E staining, Masson Trichrome staining and Sirius Red staining images of HS on day 35. Scale bar, 1 mm in H&E staining image (top), 200 μm in H&E staining image (enlarged), Masson Trichrome staining and Sirius Red staining images. **f-h** Corresponding quantitative analysis of SEI (**f**), percentage of collagen (**g**), and type I to type III collagen (**h**) of HS on day 25. (n = 3 biologically independent samples). Three independent experiments were performed and representative results are shown in **a** and **e**. Data are presented as mean ± SD and statistical significance was analyzed via one-way ANOVA with Tukey's multiple comparison test. P value: * $P < 0.05$, ** $P < 0.01$, *** $P < 0.001$.
