# Supplementary material for: Scarless wound healing programmed by core-shell microneedles
Source: Nat Commun. 2023 Jun 10;14:3431. doi: 10.1038/s41467-023-39129-6 (PMC10257705; doi:10.1038/s41467-023-39129-6)
Supplement: Supplementary file 2 — Reporting Summary [file 41467_2023_39129_MOESM2_ESM.pdf]

nature portfolio

Corresponding author(s): Zhen Gu, Yuqi Zhang, Shenqiang Wang

Last updated by author(s): May 22, 2023

Reporting Summary

Nature Portfolio wishes to improve the reproducibility of the work that we publish. This form provides structure for consistency and transparency in reporting. For further information on Nature Portfolio policies, see our Editorial Policies and the Editorial Policy Checklist.

Statistics

For all statistical analyses, confirm that the following items are present in the figure legend, table legend, main text, or Methods section.

n/a	Confirmed	
☐	☒	The exact sample size (n) for each experimental group/condition, given as a discrete number and unit of measurement
☐	☒	A statement on whether measurements were taken from distinct samples or whether the same sample was measured repeatedly
☐	☒	The statistical test(s) used AND whether they are one- or two-sided<br>*Only common tests should be described solely by name; describe more complex techniques in the Methods section.*
☒	☐	A description of all covariates tested
☐	☒	A description of any assumptions or corrections, such as tests of normality and adjustment for multiple comparisons
☐	☒	A full description of the statistical parameters including central tendency (e.g. means) or other basic estimates (e.g. regression coefficient) AND variation (e.g. standard deviation) or associated estimates of uncertainty (e.g. confidence intervals)
☐	☒	For null hypothesis testing, the test statistic (e.g. F, t, r) with confidence intervals, effect sizes, degrees of freedom and P value noted<br>*Give P values as exact values whenever suitable.*
☒	☐	For Bayesian analysis, information on the choice of priors and Markov chain Monte Carlo settings
☒	☐	For hierarchical and complex designs, identification of the appropriate level for tests and full reporting of outcomes
☒	☐	Estimates of effect sizes (e.g. Cohen's d, Pearson's r), indicating how they were calculated

Our web collection on statistics for biologists contains articles on many of the points above.

Software and code

Policy information about availability of computer code

Data collection	Olympus VS200 for histological slices, Olympus FV1000 for confocal images, Nova Nano 450 for SEM images, Beckman CytoFlex S for flow cytometry analysis, BRUKER AVIII500M for 1H NMR.
Data analysis	GraphPad Prism (version 8.2.1), Image J (version 2.1.0), FlowJo10, R package “ClusterProfiler” (version 4.0.5)

For manuscripts utilizing custom algorithms or software that are central to the research but not yet described in published literature, software must be made available to editors and reviewers. We strongly encourage code deposition in a community repository (e.g. GitHub). See the Nature Portfolio guidelines for submitting code & software for further information.

Data

Policy information about availability of data

All manuscripts must include a data availability statement. This statement should provide the following information, where applicable:

- Accession codes, unique identifiers, or web links for publicly available datasets
- A description of any restrictions on data availability
- For clinical datasets or third party data, please ensure that the statement adheres to our policy

All the other data supporting the findings of this study are available within the article and its supplementary information files and from the corresponding author upon request. Source data are provided with this paper.

Human research participants

Policy information about studies involving human research participants and Sex and Gender in Research.

Reporting on sex and gender	n/a
Population characteristics	n/a
Recruitment	n/a
Ethics oversight	n/a

Note that full information on the approval of the study protocol must also be provided in the manuscript.

Field-specific reporting

Please select the one below that is the best fit for your research. If you are not sure, read the appropriate sections before making your selection.

☒ Life sciences ☐ Behavioural & social sciences ☐ Ecological, evolutionary & environmental sciences

For a reference copy of the document with all sections, see nature.com/documents/nr-reporting-summary-flat.pdf

Life sciences study design

All studies must disclose on these points even when the disclosure is negative.

Sample size	The sample size were determined as per the pilot study or previous experimental experience and standard protocols in the field (Xiu et al., 2022, Nat Comms; Chakraborty et al., 2021, Nat Comms).
Data exclusions	No data were excluded.
Replication	The experimental findings were reliably reproduced. All of the studies are repeated at two times.
Randomization	All samples/organisms were randomly allocated into experimental groups.
Blinding	No formal blinding was used for animal studies. The investigator organizing the experimental groups and involved in sample collection was not blinded. Because the experiment results are not affected by blinding. But unbiased experimental procedure and data analysis were carried out as far as possible.

Reporting for specific materials, systems and methods

We require information from authors about some types of materials, experimental systems and methods used in many studies. Here, indicate whether each material, system or method listed is relevant to your study. If you are not sure if a list item applies to your research, read the appropriate section before selecting a response.

Materials & experimental systems

n/a	Involved in the study	
☐	☒	Antibodies
☐	☒	Eukaryotic cell lines
☒	☐	Palaeontology and archaeology
☐	☒	Animals and other organisms
☒	☐	Clinical data
☒	☐	Dual use research of concern

Methods

n/a	Involved in the study	
☒	☐	ChIP-seq
☐	☒	Flow cytometry
☒	☐	MRI-based neuroimaging

Antibodies

Antibodies used	The antibodies used for flow cytometry analysis including:<br>Brilliant Violet 605TM anti-mouse CD86 (BioLegend, catalog no. 105037, clone: PO3, lot: B349339)<br>Alexa Fluor647 anti-mouse CD206 (MMR) (BioLegend, catalog no. 141712, clone: C068C2, lot: B360876)<br>PE anti-mouse F4/80 (BioLegend, catalog no. 123114, clone: BM8, lot: B374509)<br>FITC anti-mouse CD4 (BioLegend, catalog no. 100519, clone: RM4-5, lot: B350912)

PE anti-mouse FOXP3 (BioLegend, catalog no. 126404, clone: MF-14, lot: B350965)
TruStain FcXTM PLUS (anti-mouse CD16/32 (BioLegend, catalog no. 156604, clone: S17011E, lot: B370363)
Brilliant Violet 650TM anti-mouse CD45 (BioLegend, cat: 103151, clone: 30-F11, lot: B368571
Recombinant Mouse IFN-γ (Biolegend, catalog no. 575304, lot: B324281)

The primary antibodies used for Western blot analysis including:
smooth muscle actin specific McAb (proteintech, catalog no. 67735-1-Ig, lot: 10019723)
YAP1 mouse McAb (proteintech, catalog no. 66900-1-Ig, lot: 10020242)
beta-tubulin rabbit polyAb (proteintech, catalog no. 10094-1-AP, lot: 00113489)

The antibodies used for immunofluorescence staining including:
Anti-CD86 (affinity, catalog no. DF6332)
Anti-CD206 (proteintech, catalog no. 18704-1-AP)
Anti-F4/80 (Cell Signaing, catalog no. 70076s)
Anti-α-SMA (proteintech, catalog no. 67735-1-Ig)
Anti-CD31 (R&D, catalog no. AF3628)

Validation

All antibodies are commercially available. Antibodies employed here in our manuscript were previously reported and routinely used for the application used. All companies used report quality control measures to ensure validity and reproducibility.

smooth muscle actin specific McAb (validated by proteintech)
YAP1 mouse McAb (validated by proteintech)
beta-tubulin rabbit polyAb (validated by proteintech)

Eukaryotic cell lines

Policy information about cell lines and Sex and Gender in Research

Cell line source(s)	Raw 264.7, L929 cells were purchased from ATCC.
Authentication	All of the cells are purchased from ATCC, and was not authenticated.
Mycoplasma contamination	It is negative for mycoplasma.
Commonly misidentified lines (See ICLAC register)	No commonly misidentified cells lines were used in the study.

Animals and other research organisms

Policy information about studies involving animals; ARRIVE guidelines recommended for reporting animal research, and Sex and Gender in Research

Laboratory animals	All of the male BALB/c mice were in 6-8 weeks old. All of the male New Zealand white rabbits were in 3-4 months.
Wild animals	The study did not involve wild animals.
Reporting on sex	Male mice and rabbits were used in this study.
Field-collected samples	The study did not involve samples collected from the field.
Ethics oversight	All mouse and rabbit studies were carried out under the protocols approved by the Laboratory Animal Welfare and Ethics Committee of Zhejiang University (ZJU20220079)

Note that full information on the approval of the study protocol must also be provided in the manuscript.

Flow Cytometry

Plots

Confirm that:

☒ The axis labels state the marker and fluorochrome used (e.g. CD4-FITC).

☒ The axis scales are clearly visible. Include numbers along axes only for bottom left plot of group (a 'group' is an analysis of identical markers).

☒ All plots are contour plots with outliers or pseudocolor plots.

☒ A numerical value for number of cells or percentage (with statistics) is provided.

Methodology

Sample preparation	Tissue samples were harvested (macrophages and regulatory T cells, 3 days) post-treatment. The cells were stained with antibodies post digestion to get single cells, all of the antibodies staining was followed the manufacturer's instructions.

Instrument	Beckman CytoFlex S
Software	All flow cytometry data were analyzed by FlowJo 10 software.
Cell population abundance	The instrument counts 10,000 or 50,000 cells autonomously.
Gating strategy	The gating strategies are displayed in the supplementary information.

☒ Tick this box to confirm that a figure exemplifying the gating strategy is provided in the Supplementary Information.
